# Unveiling Novel Arginase Inhibitors for Cutaneous Leishmaniasis Using Drug Repurposing and Virtual Screening Approaches

**DOI:** 10.1002/jcb.70060

**Published:** 2025-08-22

**Authors:** Eduarda Moreira Barreto, Gabriel Rodrigues Coutinho Pereira, Isadora de Salles Arêas, Júlia Mendes Fortes, Alessandra da Silva Domingos, Lucio Mendes Cabral, Carlos Rangel Rodrigues, Alessandra Mendonça Teles de Souza, Barbara de Azevedo Abrahim‐Vieira

**Affiliations:** ^1^ Laboratory of Molecular Modeling & QSAR, Faculty of Pharmacy Federal University of Rio de Janeiro Rio de Janeiro RJ Brazil; ^2^ Laboratory of Industrial Pharmaceutical Technology, Faculty of Pharmacy Federal University of Rio de Janeiro Rio de Janeiro RJ Brazil

**Keywords:** drug repurposing, *In silico*, Leishmaniasis, virtual screening

## Abstract

Leishmaniasis is a neglected tropical disease with a significant global health burden, particularly in developing countries, where it accounts for approximately 1.6 million new infections annually. Current therapeutic options are limited by severe adverse effects, toxicity, and drug resistance, highlighting the urgent need for novel treatment strategies. Arginase from *Leishmania* spp. (*Lam*ARG) has been identified as a promising therapeutic target due to its pivotal role in parasite survival and proliferation. Drug repurposing offers a strategic advantage by accelerating the identification of new therapeutics with established safety profiles, as demonstrated by repurposed agents such as miltefosine, amphotericin B, and paromomycin. This study aimed to identify FDA‐approved drugs with inhibitory potential against *Lam*ARG, leveraging structure‐ and ligand‐based computational approaches. A three‐dimensional model of *Lam*ARG was constructed through comparative modeling, followed by the compilation of known inhibitors from the literature. Molecular docking analyzed their binding interactions, generating pharmacophore hypotheses. These models were validated and applied for virtual screening of FDA‐approved compounds from the e‐Drug 3D database. Hits identified through pharmacophore‐based screening were further evaluated using molecular docking and molecular dynamics simulations to elucidate their binding modes and stability within the catalytic site of *Lam*ARG. Our findings indicate that Dabigatran exhibits strong binding affinity and key interactions within the active site of *Lam*ARG, suggesting its potential as a viable therapeutic candidate. With strong binding affinity, oral bioavailability, and a well‐established safety profile, Dabigatran emerges as a promising repurposed drug against cutaneous leishmaniasis, offering a novel, patient‐friendly therapeutic option to overcome treatment limitations and resistance challenges.

## 1 Introduction

Leishmaniasis is a neglected tropical disease that affects diverse populations in tropical and subtropical regions [1]. It is endemic in more than 90 countries, impacting approximately 1.6 million people annually [2]. Nonetheless, 90% of all globally registered cases occur in just six countries: Bangladesh, Brazil, Ethiopia, India, South Sudan, and Sudan [3]. In Brazil, leishmaniasis is considered a public health problem [4]. Beyond the social impact, leishmaniasis also places a financial burden on the Brazilian Unified Health System (SUS), which covers all medical expenses related to the diagnosis and treatment of the disease [5].

Leishmaniasis is caused by a protozoan parasite *Leishmania spp*. and is transmitted to humans through the bite of a sandfly, mainly *Phlebotomus* and *Lutzomyia* [6]. The transmission cycle of leishmaniasis varies according to the geographical region, involving a diversity of Leishmania species, invertebrate vectors, and vertebrate hosts [2].

Protozoa of the genus *Leishmania* alternate between two main morphological forms during their life cycle: amastigote and promastigote. The amastigote form is immobile and multiplies in the phagocytic mononuclear cells of vertebrate hosts. Amastigotes can be ingested by sandflies during blood feeding, reaching the vector's digestive tract, where they differentiate into motile flagellated forms, the promastigotes. The promastigotes migrate to the insect's proboscis, where they can be inoculated along with saliva during blood feeding in vertebrate hosts. Finally, the promastigotes infect the host's macrophages, where they differentiate into amastigotes, completing the parasite's life cycle [7].

The clinical spectrum of leishmaniasis is notable for its diversity and potential lethality, comprising three distinct forms: visceral leishmaniasis (VL), cutaneous leishmaniasis (CL), and mucocutaneous leishmaniasis [8]. Visceral leishmaniasis can cause systemic infection with hepatic, hematologic, and lymphatic involvement [2], with an estimated lethality rate of 9% [9]. Given its severity and high mortality, there is a growing interest in identifying novel drug targets for other clinical manifestations of the disease, particularly visceral leishmaniasis. VL remains a significant public health problem in Brazil, which accounts for approximately 96% of the cases reported in the Americas [10]. This alarming epidemiological scenario has stimulated increasing research efforts towards the discovery of new molecular targets to improve therapeutic strategies for VL [11].

Additionally, other molecular targets have been explored in different Leishmania species, expanding the therapeutic possibilities beyond arginase inhibition. For instance, ornithine decarboxylase (ODC) plays a fundamental role in the polyamine biosynthetic pathway, which is essential for parasite growth and survival, making it an attractive target for the development of new antileishmanial drugs [12]. Similarly, phosphomannomutase (PMM) has emerged as a relevant enzyme involved in the biosynthesis of glycoconjugates such as glycosylphosphatidylinositol (GPI) anchors, lipophosphoglycans, and proteophosphoglycans, all of which are involved in parasite virulence and evasion of the host immune system. Recent studies have successfully identified promising PMM inhibitors through virtual screening approaches, highlighting the potential of drug repurposing to unveil new therapeutic options for cutaneous leishmaniasis [13].

Furthermore, natural products and their derivatives continue to represent an invaluable reservoir for drug discovery against leishmaniasis. Their structural diversity and biological activities provide unique molecular scaffolds that can be applied as lead compounds [14]. A notable example is amphotericin B, a polyketide macrolide compound isolated from the actinomycete *Streptomyces nodosus*. Although initially developed as an antifungal agent, amphotericin B has been repurposed for the treatment of leishmaniasis and is currently employed, particularly in its liposomal formulation for visceral leishmaniasis, despite known limitations related to toxicity and administration [15].

Cutaneous and mucocutaneous leishmaniasis, in turn, are characterized by chronic ulcers on the skin or mucous membranes [16]. Despite its low mortality rates, the cutaneous form can cause disfiguring scars, leading to stigmatization, exclusion from community activities, and psychological disorders [17]. In Brazil, *L. amazonensis* is particularly notable for its association with the cutaneous leishmaniasis phenotype, being responsible for both diffuse anergic cutaneous (DAC) forms and cutaneous forms characterized by disseminated lesions [8].

There are no approved vaccines for human leishmaniasis, making pharmacological treatment the primary approach for disease control [18]. Nonetheless, the available drug options have several limitations, such as adverse effects, high toxicity, and long treatment duration [19]. The main therapeutic options include pentavalent antimonials, such as sodium stibogluconate (Pentostam®) and meglumine antimoniate (Glucantime®), which remain the primary choice in many countries. There are additional alternatives such as miltefosine, pentamidine isethionate, and amphotericin B. Among them, miltefosine stands out as the only option administered orally, since the other available treatments require intravenous administration, posing a significant challenge to patient compliance. Thus, the identification of new anti‐Leishmania approaches is urgently needed [8].

Arginase emerges as a promising therapeutic target in the treatment of leishmaniasis due to its importance in the metabolism of the *Leishmania* parasite, converting arginine into ornithine and urea, which contributes to the production of essential polyamines for the parasite's growth and survival within the host's cells [20]. Inhibition of the polyamine pathway has profound effects on the parasite, including mitochondrial damage, oxidative stress, and reduced viability [21]. It is worth noting that polyamine metabolic pathways in parasites differ considerably from those in vertebrate hosts, and thus, based on this difference, it is expected that new inhibitory agents will be able to selectively eliminate parasites, causing minimal, or at least tolerable, effects on the infected patient.

At the molecular level, small‐molecule inhibitors of *Lam*ARG primarily act through competitive binding within the enzyme's catalytic site. The active site contains two essential manganese ions that facilitate substrate stabilization and catalysis by coordinating with key amino acid residues [22]. Effective inhibitors mimic the structure of *L*‐arginine or its transition state, establishing direct interactions with these metal ions and residues such as His139, Asp141, Asp243, and Asp245, which are critical for catalytic function [23]. For instance, boronic acid derivatives, such as 2(*S*)‐amino‐6‐boronohexanoic acid (ABH), which act as potent competitive inhibitors by forming reversible covalent interactions with the metal centers, thereby disrupting the catalytic cycle of the enzyme [24]. Structural studies have demonstrated that inhibitors capable of forming hydrogen bonds with these key residues, as well as engaging in π‐π stacking or cation‐π interactions, tend to exhibit enhanced inhibitory potency [25]. Therefore, this pathway may represent promising targets for therapeutic intervention [26].

Drug repositioning involves identifying a new use for an already approved or experimental drug outside the scope of its original medical indication [27]. This strategy has been successfully employed by pharmaceutical industries; acetylsalicylic acid, also known as aspirin, represents one of the earliest examples of drug repositioning. Launched by Bayer in 1899 as an analgesic, aspirin was later repurposed in the 1980s, in reduced doses, as a platelet aggregation inhibitor [28]. Another example is sildenafil, initially developed as an antihypertensive drug but repurposed by Pfizer to treat erectile dysfunction and marketed as Viagra, which achieved a leading market share of 47% in the erectile dysfunction drug segment in 2012 [29]. In the specific context of leishmaniasis, drug repositioning has also proven successful, as evidenced by miltefosine, paromomycin, and amphotericin B. Miltefosine, originally an antineoplastic agent, paromomycin, an antibiotic, and amphotericin B, used for fungal infections, have all been adapted to treat leishmaniasis. This demonstrates the potential of repurposing existing drugs to provide effective treatments for this neglected tropical disease [30].

Compared to traditional drug development workflow, drug repositioning offers a faster and more cost‐effective strategy to discover new treatments for existing disorders. Drug repositioning is particularly relevant for neglected tropical diseases, where there is a pressing need for effective therapies and limited financial resources for extensive research and development. The development of new antimicrobials by the pharmaceutical industry has essentially come to a halt because it is no longer economically advantageous. Antimicrobials are used for relatively short periods and typically cure the patient, making them less profitable than drugs used to treat chronic disorders such as diabetes and psychiatric disorders [31, 32]. This trend is even more pronounced for neglected diseases like leishmaniasis, which have historically been ignored by the pharmaceutical industry due to their lower profit potential [33].

Drug repositioning campaigns can be leveraged by virtual screening (VS) strategies, a powerful *in silico* approach for identifying hit molecules [34]. VS plays a role in refining and accelerating the drug discovery process, enhancing the selection of compounds with a greater likelihood of success [35]. This approach can effectively accelerate the process of unveiling new treatments for neglected diseases through drug repositioning [36]. VS also stands out as a safe, cost‐effective, and eco‐friendly technique, aligning with the principles of the 3Rs —reduction, refinement, and replacement—which have become fundamental guidelines for minimizing animal use and promoting alternatives in scientific research worldwide [37].

Hence, we employed pharmacophore‐based virtual screening (VS) to identify potential inhibitors of *Lam*ARG within the eDrug‐3D database, which comprises FDA‐approved drugs. The compounds identified in our study hold potential for drug repositioning, offering new opportunities to enhance the quality of life for populations affected by or at risk of cutaneous leishmaniasis, which effectively addresses the urgent need for improving the available therapeutic options.

## 2 Material and Methods

### 2.1 Structural Modeling and Validation

The amino acid sequence of *Lam*ARG was obtained from UniProt (ID: O96394) [38]. The template structure for comparative modeling was identified through sequence alignment using the Protein BLAST algorithm, with the Protein Data Bank (PDB) selected as the search database and standard Protein BLAST parameters applied [39]. Sequence identity, sequence similarity, and number of alignment gaps were considered for the template selection.

The amino acid sequence of the *Lam*ARG protein was submitted to the SWISS‐MODEL server for homology modeling [40]. The crystal structure of *Lmex*ARG (PDB ID: 4IU0), identified as a suitable template using Protein BLAST, was provided via the “User Template” modeling option. Subsequently, the TM‐align server [41] was used to perform structural alignment between the template and the generated model, serving as an initial step for model validation. The theoretical 3D structure was additionally validated through quality assessment using the following algorithms: PROCHECK [42], QMEAN [43], ProSA‐Web [44], ERRAT [45], and VoroMQA [46].

### 2.2 Compilation of Known Inhibitors Targeting Lamarg

A set of inhibitors targeting *Lam*ARG was compiled through a literature review on PubMed, alongside a search in the LeishInDB [47]. Our search returned 20 inhibitors, which had their SMILES notation obtained and used to construct a _*_ *. smi* file.

Starting from the _*_ *. smi* file containing the 20 inhibitors, we generated their three‐dimensional structures using the OpenBabel 3.1.1 software [48]. The ionized state at pH 7.4 was considered to add hydrogens to the molecules. This pH value is found in *Leishmania* amastigotes forms, which maintain their internal pH close to neutral, contrasting with the external acidic environment [49]. The structures were, then, minimized using the MMFF‐94s force field [50].

### 2.3 Molecular Docking

A redocking simulation was applied to validate the docking protocol within the active site of *Leishmania spp*. arginase [51]. The GOLD software 2022.3.0 [52] was used for the (re)docking. Given that the structure of *Lam*ARG has not been experimentally resolved to date, we initially performed a re‐docking within the active site of *Lmex*ARG complexed with ABH, which corresponds to the PDB code 4IU0 [22]. *Lmex*ARG shares a 99.4% sequence identity with *Lam*ARG, with a highly conserved amino acid profile, especially within the active site.

The protein structure was initially prepared by removing non‐essential heteroatoms, including co‐crystallized ligands and solvent molecules. Hydrogen atoms were then added, and protonation states were assigned using the internal algorithm of the GOLD software [52]. Ligand structure, i.e., ABH, were prepared in OpenBabel 3.1.1 [48], considering a pH of 7.4 [49], and subsequently energy‐minimized using the MMFF94s force field. For the docking simulations, the prepared protein structure was saved in PDB format, and the ligand was supplied in SDF format.

The molecular docking studies were conducted using the genetic algorithm (GA) available in the GOLD software, which was selected to explore ligand conformations [53]. Among the scoring functions available in GOLD 2022.3.0, the ChemScore function was selected due to its ability to effectively estimate the contributions of metal–ligand bonds to the binding free energy [52]. It is particularly important in the context of the arginase active site, which includes two catalytic Mn^2+^ ions [22]. The active site was defined by centering a grid box with dimensions of 10 Å on the ligand, which corresponds to coordinates: x = −9.328; y = −22.064; z = 8.027.

The simulations were performed using the semiflexible approach, considering the ligand as flexible and the receptor as rigid. The conformational search was conducted with 100 runs of the genetic algorithm, using the program's default parameters. Early stopping of the molecular docking simulations was configured to occur when the top three ChemScore solutions differ by less than 1.5 Å. Finally, the solution with the highest ChemScore was selected to represent the simulation.

The *root‐mean‐square deviation* (RMSD) values obtained from aligning the predicted and experimental poses were considered for re‐docking validation, from which RMSD values < 2 Å indicate accurate predictions [54]. Interactions were additionally analyzed through visual inspection of the 3D docked complex using Pymol 2.5.0 software [55].

After redocking validation, the *Lam*ARG structure was aligned with *Lmex*Arg, and then molecular docking was carried out on the aligned *Lam*ARG structure with the 20 arginase inhibitors, following the same methodology previously described for re‐docking.

### 2.4 Pharmacophore Modeling and Validation

The structure of the *Lam*ARG complex with docked inhibitors was submitted to the Pharmit server to generate pharmacophore hypotheses [56]. Following this, a chemical compound library, referred to as the test set, was constructed to validate the generated maps. To achieve this, decoys—molecules with similar properties to the active compounds but not expected to bind to the target receptor [57] —were generated. The active compounds previously compiled were submitted to the DUD‐E server (https://dude.docking.org/), which returned up to 50 decoys for each ligand [58]. SMILES notations for the test set molecules were used as input (*. smi) to construct conformer libraries on the Pharmit server [56]. Finally, the pharmacophore hypotheses generated were validated using the test set.

The standard parameters from the Pharmit server were used for the validation screening of the test set. The screening process incorporated the following shape constraints: excluded shape for the receptor and included shape for the ligand [56]. The predicted classes, i.e., active or inactive, were compared to the experimentally determined classes. The predictive performance of the pharmacophore hypotheses was then evaluated using the following classification metrics: recall, specificity, sensitivity, enrichment factor (EF), goodness‐of‐hit score (GH), Matthew's correlation coefficient (MCC), and F1‐score. The model demonstrating the highest predictive performance was selected as the valid pharmacophore hypothesis [59].

### 2.5 Virtual Screening

The e‐Drug 3D database encompasses data on 2083 FDA‐approved drugs, detailing their pharmacological effects, available pharmaceutical formulations, and therapeutic targets [60]. Their corresponding SMILES notation was stored in a _*_ *. smi* file and submitted to the Pharmit server [56] to construct a conformer library, henceforth referred to as the e‐drug library. The validated pharmacophore hypothesis of *Lam*ARG was selected for virtual screening (VS) against the e‐drug library.

Drugs that exhibited a significant match to the pharmacophore hypothesis and were not classified as prodrugs—compounds that require metabolic conversion to become active [61] —had their binding modes within the *Lam*ARG active site elucidated through docking simulations. These simulations were conducted following the docking protocol previously validated through *redocking* using the GOLD v5.7.0 software [52]. Finally, the predicted binding modes were verified through visual inspection in 3D using PyMOL software, ensuring a comprehensive assessment of the interactions between the drugs and their potential target [55].

Given that the virtual screening (VS) was conducted within the framework of drug repurposing, drugs not orally administered were filtered out from the screening process. This approach ensures that the identified candidates are more likely to be viable for repurposing as oral medications, thereby improving patient adherence to the treatment [34].

### 2.6 Molecular Dynamics Simulations

Independent molecular dynamics (MD) simulations were conducted for the *Lam*ARG protein complexed with either Protokylol or Dabigatran, using GROMACS 2020.6 [62]. The initial structures for the simulations were derived from the docking predictions of the corresponding *Lam*ARG complexes. The ligands were prepared using the ACPYPE server to generate AMBER‐based topologies [63]. The AMBER99SB force field was selected for the simulations, using the TIP3P water model to solvate the protein‐ligand complexes in a triclinic box, resulting in the addition of 9856 water molecules. The protein was centered within the box, with a margin of 1.0 nm from its surface, ensuring adequate solvation inside a system with dimensions of 5.868 × 4.868 × 4.536 nm.

To neutralize the system's charge, 32 Na⁺ and 37 Cl⁻ ions were added at a concentration of 0.15 mol/L. Energy minimization was performed using the steepest descent method until convergence was achieved, followed by equilibration in both NVT (constant number of particles, volume, and temperature) and NPT (constant number of particles, pressure, and temperature) ensembles. The NVT phase was conducted for 100 ps at 310 K using the v‐rescale thermostat, while the NPT phase was carried out for 100 ps at 1 bar and 310 K with the Parrinello‐Rahman barostat [64].

After system equilibration, 300 ns of production MD simulations were conducted. The Particle Mesh Ewald (PME) method was used to handle long‐range electrostatic interactions [65], and the LINCS algorithm was applied to constrain all covalent bonds. A time step of 2 fs was used for the simulations [66].

Trajectory analysis was performed with the following GROMACS tools: *gmx rms*, *gmx sasa*, *gmx gyrate*, and *gmx mindist*. The RMSD was calculated for the ligands and protein backbone atoms. The radius of gyration (Rg) and solvent‐accessible surface area (SASA) were computed from the protein atoms. After confirming system equilibration by assessing the stability of RMSD, Rg, and SASA over time, a more detailed analysis of the interaction profile was conducted, focusing specifically on the equilibration phase of the simulation [67].

The number of protein‐ligand contacts and the minimum distances between these groups were calculated using a 5 Å cut‐off [67]. Visual Molecular Dynamics (VMD) 1.9.3 [68] was employed to calculate the hydrogen bonds formed between LamARG and the ligands, along with their respective occupancies throughout the simulations. The analysis considered geometric cut‐off criteria, specifically bond angles ≤ 30° and interatomic distances ≤ 3.5 Å [67].

Finally, the binding free energy calculations (ΔG_binding)_ were performed using the gmx_MMPBSA software [69], which implements the Molecular Mechanics Generalized Born Surface Area (MM/GBSA) method. This approach incorporates molecular mechanics potential energies (E_MM_) alongside the polar (E_GB_) and nonpolar solvation (E_SA_) components.

Visualization and descriptive statistical analyses were performed using Python scripts that integrated the gmx_MMPBSA workflow [69] with functions from the *matplotlib*, *seaborn*, *pandas*, and *numpy* libraries [70].

## 3 Results and Discussion

### 3.1 Structural Modeling and Validation

**Figure 1:**
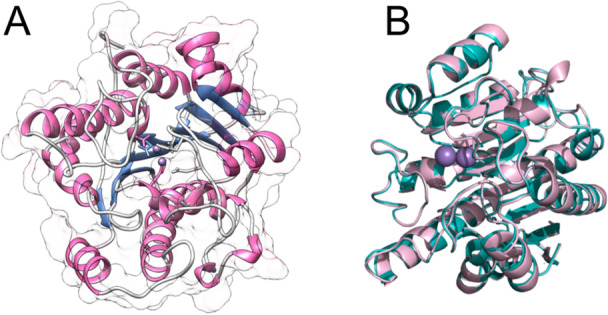
Three‐dimensional structure of the *Lam*ARG model and structural alignment. (A) Theoretical model of *Lam*ARG generated using comparative modeling. Pink arrows represent beta‐sheet regions, and blue helices indicate alpha helix regions. Additionally, the protein's surface is depicted in the figure. (B) Structural alignment between the *Lam*ARG structure (depicted in blue) and the template structure (depicted in green).

The three‐dimensional structure of *Lam*ARG has not yet been experimentally determined [71]. Therefore, comparative modeling was performed to construct a theoretical model (Figure 1A) using the crystallographic structure of *Lmex*ARG complexed with the inhibitor ABH (PDB code 4IU0) as a template. This template was selected due to its high sequence identity with the target sequence (*Lam*ARG), i.e., 99.4%, which substantially exceeds the typical threshold of 30% required for generating accurate models through comparative modeling [72].

Additionally, the alignment E‐score provided by Blastp, which reflects the degree of identity between the target sequence and queried sequences, presented an “E” value close to zero. This result indicates a significant match with the selected template [73].

Based on the selected template, a complete model of the *Lam*ARG protein was successfully generated (Figure 1A), which proceeded to the subsequent step of structural validation.

A critical aspect of evaluating the quality of protein models generated through comparative modeling involves comparing the predicted structure with its reference template to quantify structural similarities. Structural similarity can be assessed by aligning the theoretical model with the reference template and measuring the distances between their corresponding atom pairs [74]. Therefore, to ensure structural similarity between the theoretical model of *Lam*ARG and its reference template, an alignment was performed on the TM‐align server, which provided distinct similarity metrics: RMSD and TM‐score [41].

The model exhibited an RMSD value of 0.29 Å and a TM‐score of 0.99 relative to the reference template structure, meeting the established thresholds for identifying structurally related proteins, i.e., RMSD < 2 Å and TM‐score > 0.5 [75]. A visual inspection of the alignment shown in Figure 1B reaffirms the structural similarity between the theoretical *Lam*ARG model and the crystallographic fragment of human *Lmex*ARG.

**Figure 2:**
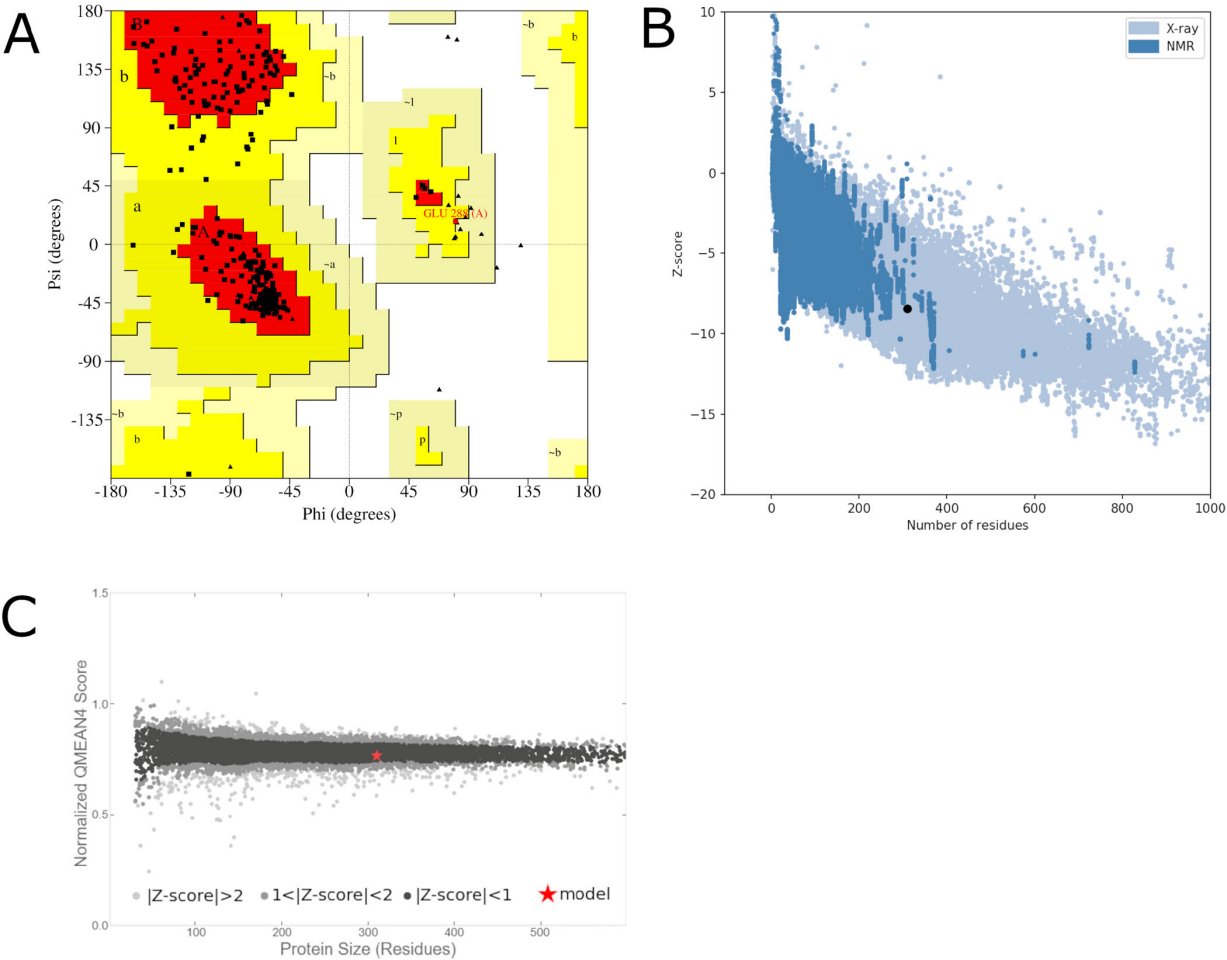
Structural validation of the theoretical model of *Lam*ARG using Procheck, ProSa and QMEAN. (A) Ramachandran plot generated from the validation on the PROCHECK server. Protein residues are depicted as black squares and triangles. Each residue is plotted into distinct regions of the Ramachandran plot based on the distribution of its phi and psi angles, as follows: most favored (red), additionally allowed (yellow), generously allowed (beige), and disallowed regions (white). (B) Structural validation on the ProSa‐web server. The X‐axis represents the number of amino acids in the protein, while the Y‐axis represents the Z‐score estimated for the model. The submitted structure is denoted by a black dot, with lighter blue indicating structures resolved by X‐ray crystallography and darker blue indicating those resolved by NMR. (C) Structural validation on the QMEAN server. The X‐axis represents the protein length considering the number of amino acids, while the Y‐axis depicts the Z‐score estimated for the model. The QMEAN scores of experimental structures are categorized into quality bands based on their Z‐scores, shown in shades from black to gray. The QMEAN score of the submitted structure is indicated by a red “x”.

Additionally, the overall quality of the model was evaluated using validation algorithms, starting with PROCHECK, which assessed its stereochemical quality based on the distribution of phi and psi angles within the Ramachandran plot. Structural validation depends on the number of residues allocated in the most favored regions of the plot. High‐resolution protein structures typically have more than 90% of their amino acids in these regions [76]. The theoretical *Lam*ARG model exhibited 90.9% of residues in the most favorable regions (Figure 2A), aligning with expected values for high‐resolution protein structures.

ProSa‐Web evaluates the overall quality of protein structures by analyzing their potential energy based on the three‐dimensional structure, producing a Z‐score as a general quality metric. The tool generates a distribution of Z‐scores using crystallography and NMR‐based protein structures from the PDB, which it then plots in a graph. This graph illustrates the expected distribution of Z‐scores observed in experimentally determined structures. ProSa‐Web subsequently calculates and integrates the Z‐score of the target protein model into this graphical representation [44]. When comparing the estimated Z‐score for the theoretical *Lam*ARG model, i.e., −8.45, its overall quality score falls within the empirical distribution of crystallographic structures from the PDB (Figure 2B).

QMEAN integrates the analysis of various structural features, including atomic contacts, torsion angles, solvation potential, and secondary structure, to generate a comprehensive score known as the QMEAN‐score, evaluating the overall quality of a submitted model. Similar to ProSa‐Web, QMEAN calculated an empirical distribution of QMEAN‐score values based on data from 9766 experimentally determined protein structures from the PDB. By plotting the estimated QMEAN‐score for the target model against this distribution [43], QMEAN determines whether the model aligns with established standards. As shown in Figure 2C, the predicted overall quality score for the *Lam*ARG model, i.e., 0.77, falls within the range of QMEAN scores observed in the empirical distribution of high‐resolution protein structures.

**Figure 3:**
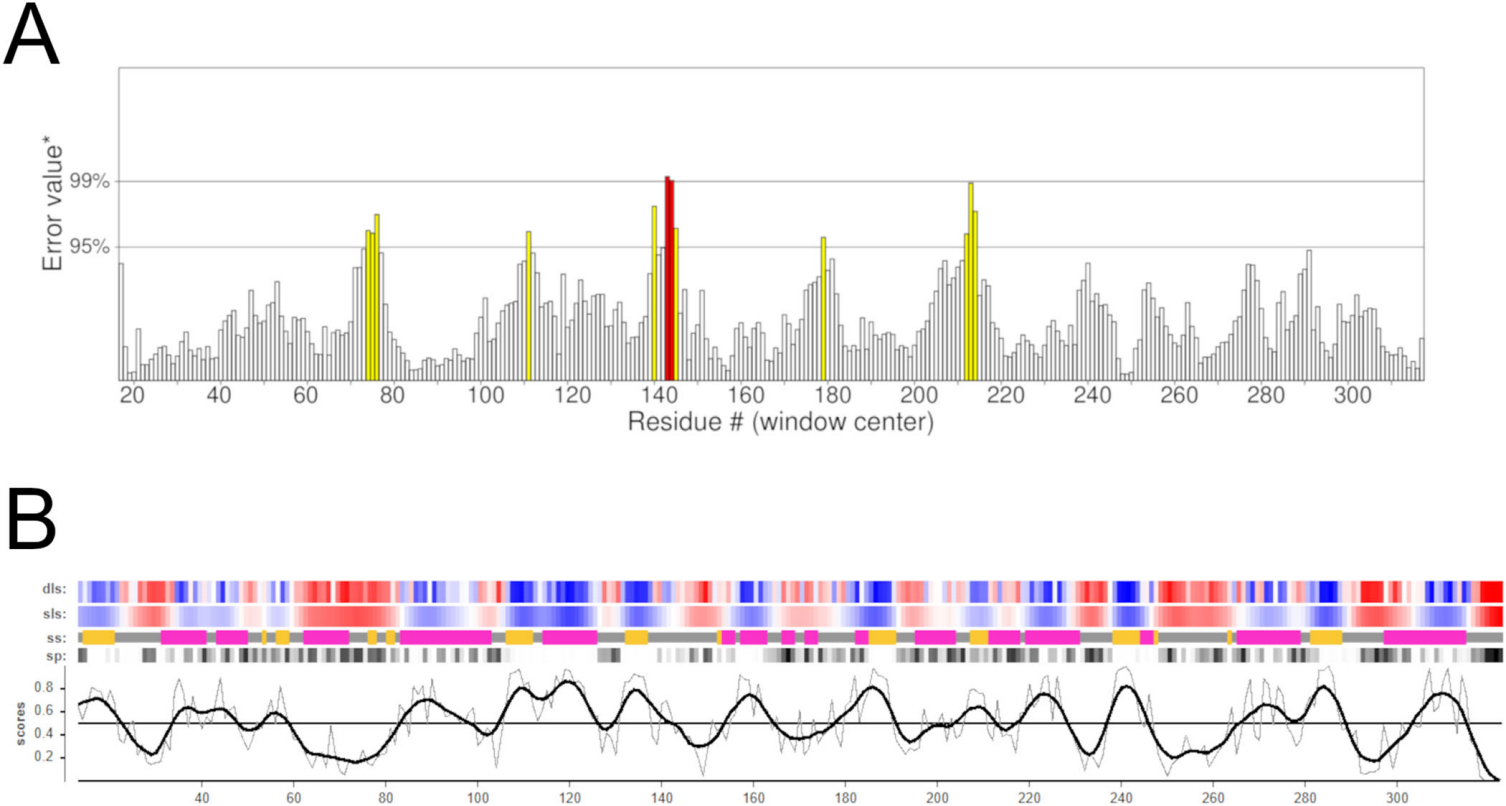
Validation of the theoretical model of *Lam*ARG using ERRAT (A) and VoroMQA (B). (A) Error value plot generated by ERRAT relative to each amino acid position. Amino acid positions are displayed on the X‐axis, while error values are shown on the Y‐axis. *Two lines on the error axis indicate the confidence levels for rejecting regions based on their error values, which corresponds to 95% and 98% confidence levels, respectively. (B) VoroMQA validation plot, displaying local scores as thin gray lines, and smoothed scores as thick black lines. Detailed local score (dls), smoothed local scores (sls), secondary structure (ss), solvent accessible surface percentage (sp) are also presented for better comparison. Dls and sls are color‐coded from red to blue to indicate quality from lowest to highest, while ss are color‐coded by type: pink for α‐helices, yellow for β‐sheets, and gray for coils. In turn, sp is color‐coded from white to black to indicate the range from lowest to highest values.

ERRAT identifies potentially erroneous regions in protein structures by analyzing their nonbonded interaction distances. It estimates the probability of error for each amino acid based on the empirical distribution of nonbonded contacts derived from a subset of the PDB comprising 96 high‐resolution protein structures. Typically, high‐resolution protein structures exhibit error probabilities below 5% for approximately 95% of their residues [45]. The theoretical *Lam*ARG model demonstrated that 96.01% of its residues fall within this expected error range (Figure 3A). VoroMQA (Model Quality Assessment based on Voronoi Diagram) evaluates the overall quality of protein structures by analyzing interatomic interactions. It calculates local scores for each amino acid to estimate the overall quality of the target structure. Based on the analysis of an empirical distribution derived from the PDB, VoroMQA determined that most high‐resolution protein structures typically achieve global quality scores of ≥ 0.4. The theoretical LamARG model presented a global quality score of 0.56 (Figure 3B), reaffirming its validity [46].

Overall, the model met the necessary validation cut‐off ranges for all the algorithms investigated (Figures 1, 2, 3), demonstrating quality comparable to experimentally determined protein structures. Additionally, the model exhibited significant similarity to its reference structure, i.e., PDB code 4IU0. Therefore, it was considered validated and selected for subsequent analyses.

### 3.2 Compilation of Known Inhibitors Targeting Lamarg

In total, 20 known inhibitors of *Lam*ARG from different structural classes were compiled from the literature and database, including: i) phenolic acids: caffeic acid (**1**) [77], gallic acid (**2**) [25]; ii) flavonoids: catechin (**3**) [25], epigallocatechin‐3‐gallate (**4**) [25], quercetin (**5**) [77], **6** [78], **7** [25], **8**, **9**, **10**, **13**, **14**, **15**, **16**, **17**, **18**, **19**, **20** [79]; iii) lignans: **11**; iv) diterpenoids: **12** [79].

**Figure 4:**
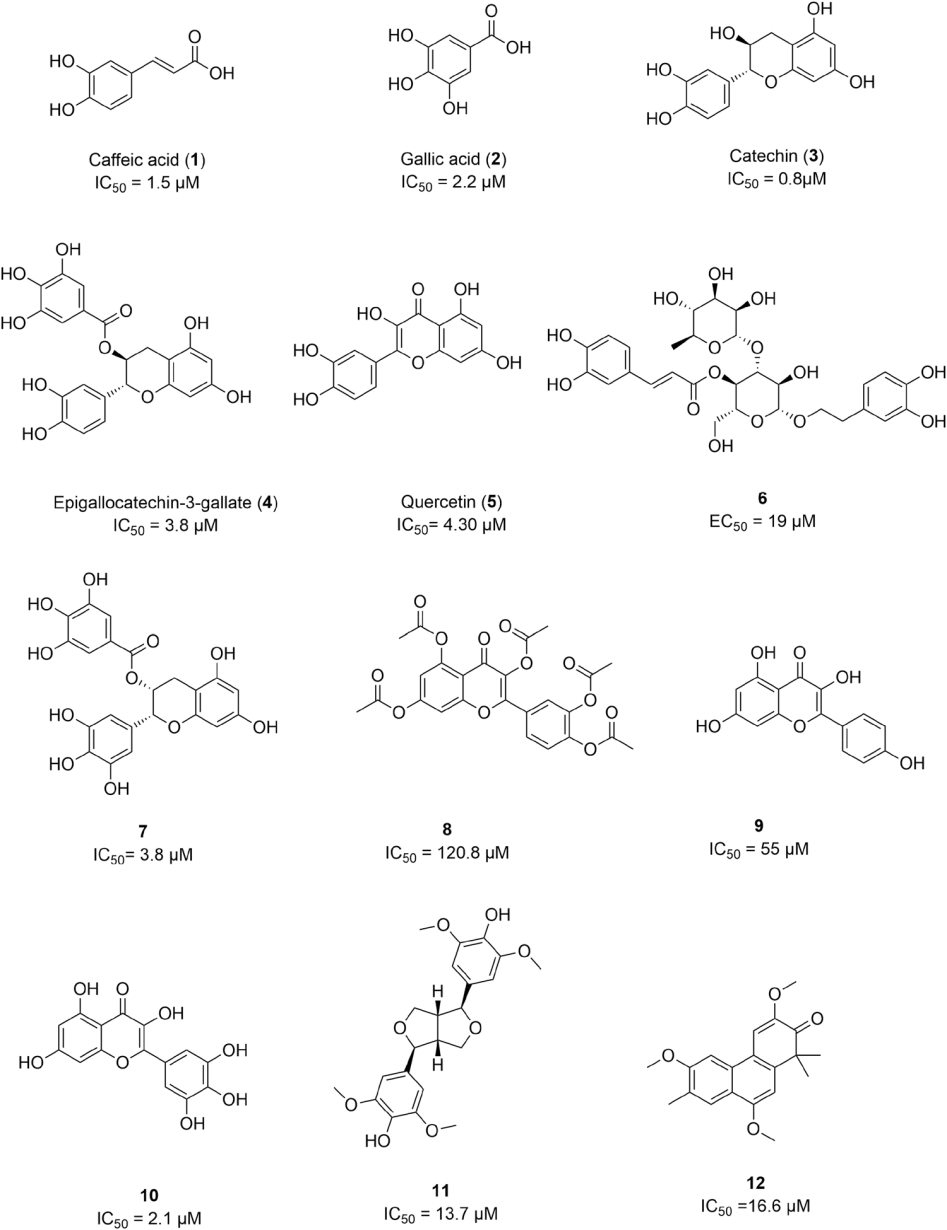
Compounds with activity against *Lam*ARG compiled from the literature (part 1).

**Figure 5:**
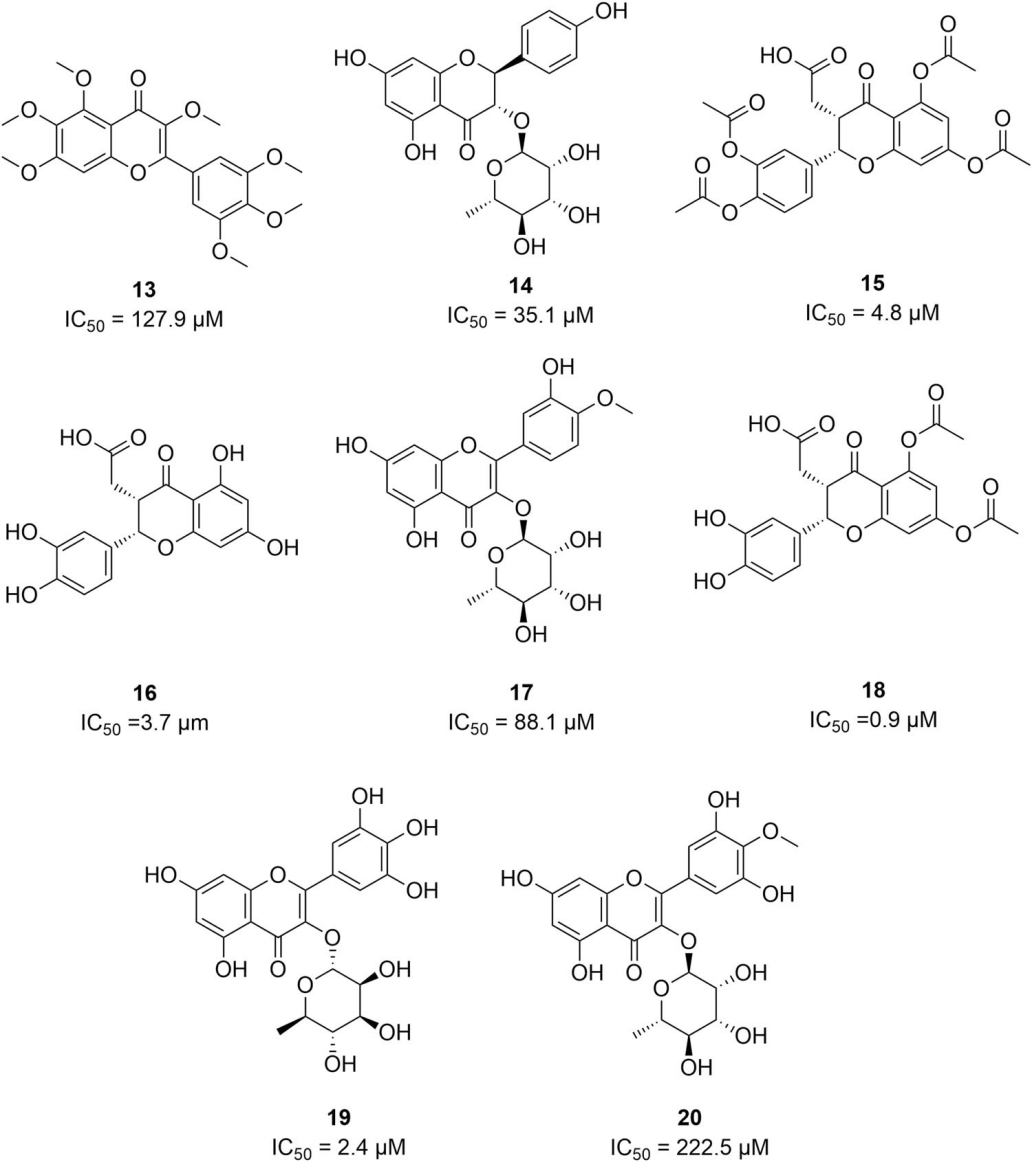
Compounds with activity against *Lam*ARG compiled from the literature (part 2).

The chemical structures of these *Lam*ARG inhibitors are displayed in Figures 4 and 5 along with their respective IC_50_ values, providing a visual representation of their molecular diversity and inhibitory potency.

### 3.3 Molecular Docking

*Lmex*ARG exhibits a remarkable 99.4% sequence identity with *Lam*ARG, demonstrating a highly conserved amino acid composition, particularly in the active site. Since the structure of *Lam*ARG has not yet been experimentally determined and no structures of *Lam*ARG complexed with ligands are available [80], a direct redocking analysis was not possible. Therefore, we conducted the redocking analysis using *Lmex*ARG before proceeding with docking studies on the modeled structure of *Lam*ARG.

As previously demonstrated by our group, the conserved core region shared among *Leishmania spp*. arginase proteins are expected to yield similar binding interactions. This establishes *Lmex*ARG as a valuable reference for investigating ligand binding profiles in our study, offering a robust foundation for predicting how ligands may interact with the active site of the yet uncharacterized receptor, *Lam*ARG. Thus, insights gained from the redocking analysis of LmexARG were effectively leveraged to enhance the reliability of subsequent docking studies on this novel target structure [80].

**Figure 6:**
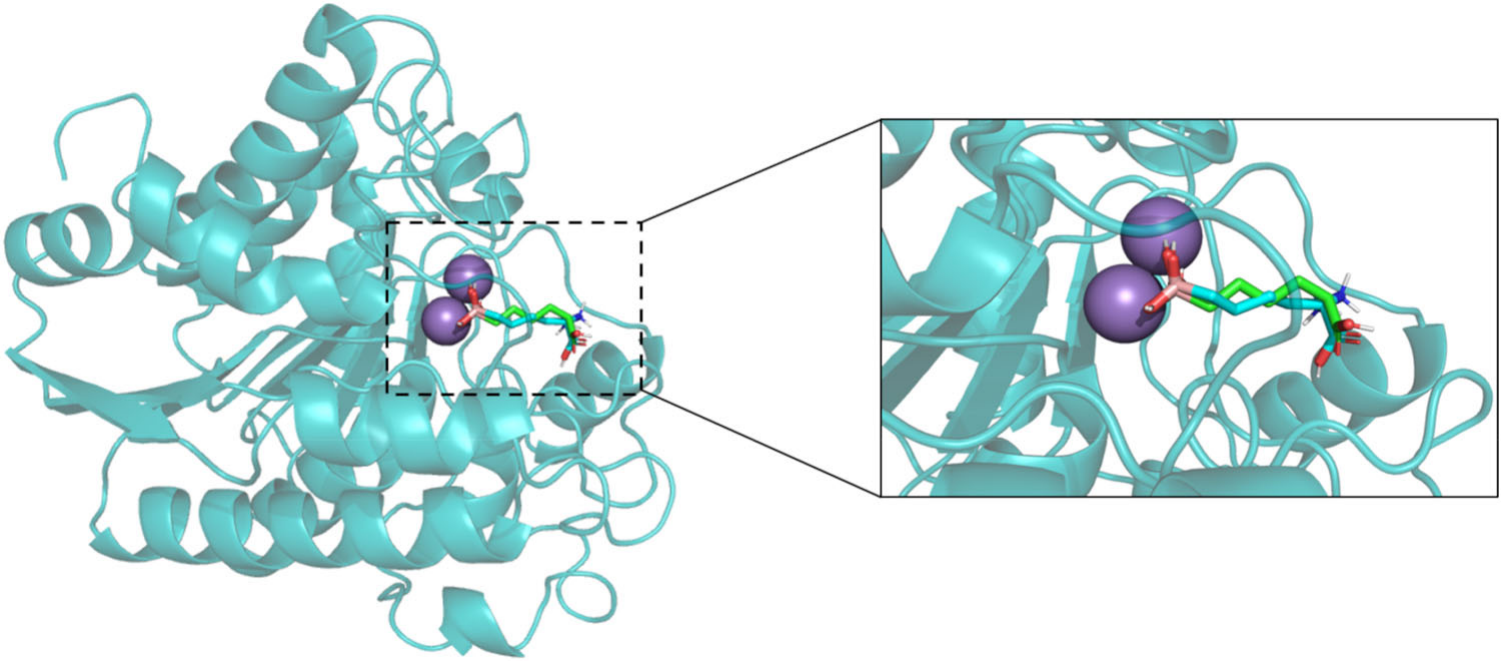
Superposition of the crystallographic pose of the ligand 2(s)‐amino‐6‐borohexanoic acid (ABH) with the predicted conformation from redocking. The crystallographic pose, obtained from PDB code 4IU0, is shown in green, while the pose predicted by the GOLD v5.7.0 software is shown in cyan. The protein's three‐dimensional structure is depicted in cartoon style and colored ocean blue.

The redocking analysis yielded an RMSD of 0.80 Å when comparing the predicted pose with the crystallographic binding pose. A root‐mean‐square deviation (RMSD) cut‐off of 2.0 Å was established as the validation threshold to assess the reliability of the redocking procedure. This cut‐off ensures that the predicted ligand conformation closely aligns with the experimentally determined structure. Visual inspection of the interactions, as shown in Figure 6, further confirmed this alignment, reinforcing the reliability of the docking protocol for accurately modeling ligand binding within the active site of *Leishmania spp*. arginase [51].

Following the redocking validation, molecular docking simulations were conducted on 20 known *Lam*ARG inhibitors. The docking results are presented in Figures S1 to S3 and summarized in Table 1. Ionic interactions were evaluated but not included in Table 1, as none were identified in the docking results.

**Table 1:** Docking affinity scores and predicted binding modes for known inhibitors of *Lam*ARG interacting with the target protein.

Compound	Activity (µM)^a^	Score	Hydrogen bond	π‐π	Cation‐π	Ion‐dipole	Salt bridge
Caffeic acid **(1)**	1.5	29.97	N143	—	—	Mn^+2^	H139
Gallic acid **(2)**	2,2	27.27	D245, D243, H154, D141	—	—	Mn^+2^	H154
Catechin **(3)**	0,8	29.69	D141, E197, D243, N143	H139	—	Mn^+2^	—
Epigallocatechin‐3‐Gallate **(4)**	3,8	26.94	G256, H154, N143	—	—	Mn^+2^	—
Quercetin **(5)**	4.30	27.68	D245, A192	H139	—	Mn^+2^	
**6**	19^b^	20.82	D194, D143, D141, N152, D150, Asp243	H139	—	Mn^+2^	—
**7**	3.8	26.79	D245, D141, N152, D194	H154	—	Mn^+2^	—
**8**	120.8	18.19	D152	H139	—	Mn^+2^	—
**9**	55	22.05	S150, T148, N152	—	H154	—	—
**10**	22.1	26.24	D245, D141, N152	H154	—	Mn^+2^	—
**11**	1.6	17.61	D141, H154	H139	H139	—	—
**12**	16.6	19.59	N152	H139	—	—	—
**13**	127.9	17.88	H139, N152, H154	H139	—	—	—
**14**	35.1	19.18	D194, H139	H154	—	—	—
**15**	4.8	14.19	N152, S150, V149	—	H139	—	—
**16**	3.7	29.80	D245, D243, N152, A192	H139	—	Mn^+2^	H154
**17**	88.1	16.89	N152, D141, N143, H154	—	—	—	—
**18**	0.9	29.38	D243, D141, H154	H139	—	Mn^+2^	—
**19**	2.4	18.93	D194, V149, D141, G155, N143, H154	—	—	—	—
**20**	223.5	15.17	His154, D152, D141, D194, N143	—	—	—	—

^a^ Experimental activity as reported in the literature.

^b^ The activity of all compounds is expressed as IC₅₀, except for compound **6**, for which the activity is reported as EC₅₀.

The enzymatic activity of *Lam*ARG relies on key residues that facilitate substrate binding and coordinate with metal ions. Specifically, residues His139, Asp243, and Asp245 coordinate with one Mn²⁺ ion, while His114, Asp137, and Asp141 coordinate with another Mn²⁺ ion. In turn, His154 and Glu288 are known to be directly involved in substrate binding, i.e., arginine. Further literature indicates that residues Asp194 and Thr257 may also play an important role for optimizing selective inhibitors [24]. In addition to Glu288, the catalytic triad of *Lam*ARG is formed by Asp141, which stabilizes the hydroxide ion, and His139, which function as a proton carrier [23].

Thus, the results, summarized in Table 1, revealed that these inhibitors likely exert their effects through two main mechanisms: (I) Competing with the natural substrate: Binding to the active site and preventing arginine binding; (II) Disrupting the catalytic mechanism: Interfering with Mn^+2^ coordination or the interactions within the catalytic triad.

Among the compounds analyzed, **18** stood out with one of the lowest IC₅₀ values (0.9 µM), indicating a strong inhibitory activity. This high potency is likely attributed to the compound's predicted interactions with several key residues in the catalytic site of *Lam*ARG, including Asp243, Asp141, His154, and His139. These interactions are thought to contribute to stabilizing the ligand within the enzyme active site, suggesting a relation between the number of hydrogen bonds formed and the compound's experimental inhibitory activity. Additionally, other compounds, such as gallic acid, **11**, and **7**, demonstrated interactions with key residues of the *Lam*ARG protein, particularly those involved in the catalytic triad, i.e., His139, Asp141, and Glu288 [23]. By targeting critical sites, these compounds may effectively modulate the activity of *Lam*ARG, further contributing to their promising inhibitory effects.

In contrast, compounds **8** and **9** exhibited significantly higher IC₅₀ values of 120.8 µM and 55 µM, respectively, indicating poor inhibitory potency. These compounds were predicted to form a limited number of hydrogen bonds with key residues, potentially due to unfavorable positioning within the active site. For instance, compound **8** formed only one hydrogen bond with Asn152 and engaged in π‐π interactions with His139, while compound **9** primarily established three hydrogen bonds with Asn152, Thr148, and Ser150. This suggests that the reduced number of interactions formed by these compounds may lead to lower stability within the catalytic site, ultimately resulting in decreased inhibitory efficacy.

### 3.4 Pharmacophore Modeling and Validation

Pharmacophore mapping identifies key steric and electronic features required for optimizing intermolecular interactions with a receptor, thereby triggering or blocking its biological activity. In this approach, the chemical groups that govern protein‐ligand interaction are represented in three dimensions, including hydrogen bond donors or acceptors, hydrophobic or aromatic interactions, and charged groups. Additionally, exclusion volumes can be incorporated to mimic the steric constraints of the binding site, enhancing the specificity and accuracy of the pharmacophore model [81].

To ensure the reliability of the pharmacophore map, it is mandatory to evaluate its predictive performance using a well‐defined test set comprising both active and inactive compounds for the receptor of interest [82]. Therefore, before developing the actual pharmacophore map, a chemical compound library was created to serve as a test set for validating the generated maps.

The test set was composed of 20 active *Lam*ARG inhibitors compiled from the literature and 1,000 decoys retrieved from the DUDE search. Nonetheless, five decoys were considered invalid by Pharmit, totaling 1015 compounds.

The following metrics were computed from the evaluation of the test set to assess the predictive performance of the pharmacophore hypotheses generated for the known *Lam*ARG inhibitors [83]:

- *Total hits:* Ht *;*
- *Active hits* or True Positive (TP): Ha;
- Total number of actives: A *;*
- Total number of compounds: D *;*
- Enrichment factor: $\frac{\left(\mathrm{Ha x D}\right)}{\left(\mathrm{Ht x A}\right)}$ *;*
- Goodness*‐of‐hit score:* $\left(\frac{\mathrm{Ha x}\;\left(3A+\mathrm{Ht}\right)}{4\mathrm{HtA}}\right)x\;\left(1-\frac{\left(\mathrm{Ht}-\mathrm{Ha}\right)}{\left(D-A\right)}\right)$ *;*
- True negatives (TN):D−Ht−A+Ha *;*
- False negatives (FN): A−Ha *;*
- False positives (FP): Ht−Ha *;*
- Matthew's correlation coefficient*:* $\frac{\mathrm{TPxTN}-\mathrm{FPxFN}}{\sqrt{\left(\mathrm{TP}+\mathrm{FP})x(\mathrm{TP}+\mathrm{FN})x(\mathrm{TN}+\mathrm{FP})x(\mathrm{TN}+\mathrm{FN}\right)}}$.

The detailed results are presented in Table 2, showcasing the performance of the pharmacophore models. Inhibitors that did not produce valid pharmacophore hypotheses in Pharmit were subsequently excluded from further consideration, as they were unlikely to contribute to the identification of effective drug candidates.

**Table 2:** The performance of the pharmacophore models on the test datasets.

Hypothesis	Pharmacophore	GH	EF	MCC
Epigallocatechin 3‐gallate	HD (6.2, −23.7, 7.2); HD (10.1, −19.9, 15.4); HD (10.1, −19.9, 15.4); HD (15.8, −22.4, 6.7); HA (5.5, −21.1, 7.3)	0.78	8.42	0.39
Compound 8	HA (7.13, −20.52, 7.96); HA (13.65, −24.99, 16.2); AR (10.75, −21.55, 8.95)	0.03	44.94	0.15
Compound 9	HD (16.23, −24.72, 8.91); HD (11.01, −25.25, 8.6); HA (16.23, −24.72, 8.91); AR (8.77, −22.11, 8.91)	0.10	25.27	0.22
Compound 11	HD (6.2, −21.44, 7.58); HA (10.79, −22.87, 10.61); AR (8.94, −21.82, 7.59)	0.01	47.75	0.10
Compound 12	HD (12.31, −23.66, 11.58); HA (11.02, −22.15, 13.4); AR (12.15, −16.7, 10.49); AR (12.7, −18.97, 9.68)	0.03	11.23	0.05
Compound 13	HA (11.9, −23.6, 11.6); HA (13.0, −19.1, 7.0)	0.02	50.55	0.13
Compound 14	HD (18.3, −22.1, 7.7); HA (12.6, −19.8, 6.9)	0.01	33.70	0.05
Compound 15	HA (16.4, −25.0, 8.8); HA (12.1, −23.3, 11.7); AR (12.2, −21.6, 8.4)	0.01	36.51	0.05
Compound 17	HD (9.9, −22.5, 7.5); HD (13.4, −24.3, 8.1); HD (13.8, −24.3, 8.1); HA (11.1, −23.0, 12.5)	0.08	16.85	0.15
Compound 18	HD (5.7, −23.2, 7.5); HD (6.1, −20.5, 7.2); HA (11.3, −22.8, 11.1); HA (6.1, −20.5, 7.2); AR (8.2, −22.1, 7.9)	0.86	25.27	0.68

*Note:* The compounds not listed in the table did not yield results on the Pharmit server.

Abbreviations: AR, aromatic; EF, enrichment factor; GH, goodness‐of‐hit score, HA, hydrogen acceptor; HD, hydrogen donor; MCC, Matthew's correlation coefficient.

Among all the generated hypotheses, only the one generated from compound **18** was validated as a pharmacophore through the analysis of goodness‐of‐hit (GH), enrichment factor (EF), and Matthew's correlation coefficient (MCC), which are well‐established metrics for this purpose [59]. The GH score was employed to ensure the model's ability to accurately identify active compounds during the screening process [84]. This metric ranging from 0 to 1, with a score of 1 indicating a perfect classifier [85]. GH values exceeding 0.6, such as the 0.86 obtained for the hypothesis of compound 18 in this study (Table 2), suggest that the model is effective in identifying hit compounds (actives), thereby validating these hypotheses [86].

The analysis of EF was also assessed, providing further insight into the model's effectiveness in prioritizing potentially active compounds [87]. For the hypothesis of compound 18, the computed EF score was 25.27, indicating an effective enrichment capacity, i.e., the ability to identify active compounds relative to a random selection of compounds from the dataset. data set. Nonetheless, this metric is directly influenced by the number of observations in each class. Therefore, the enrichment factor calculated for the models in this study may not accurately represent the model's ability to identify compounds of interest, as the predominance of a specific class can mask the model's effectiveness in identifying active compounds [87].

Considering this, MCC values were additionally evaluated, providing an even more robust measure of model performance, particularly in unbalanced datasets. This metric takes into account all four quadrants of the confusion matrix, thereby offering a comprehensive assessment of the model's predictive ability across both positive and negative classes [88]. MCC can range from −1 (worst performance) to +1 (best performance), where values lower than zero indicate worse predictive capability than a random model, and values greater than zero indicate better predictive capability than random. Notably, only the hypothesis of compound 18 demonstrated an MCC value exceeding 0.5, thus, indicating a positive correlation between predictions and actual classes [89].

Therefore, the pharmacophore model derived from compound 18 exhibited satisfactory performance metrics during validation, demonstrating its ability to efficiently identify active compounds (hits) while distinguishing them from inactive ones. This suggests that the model is well‐suited for screening compounds with potential biological activity against *Lam*ARG, thereby facilitating the identification of potential therapies for cutaneous leishmaniasis.

**Figure 7:**
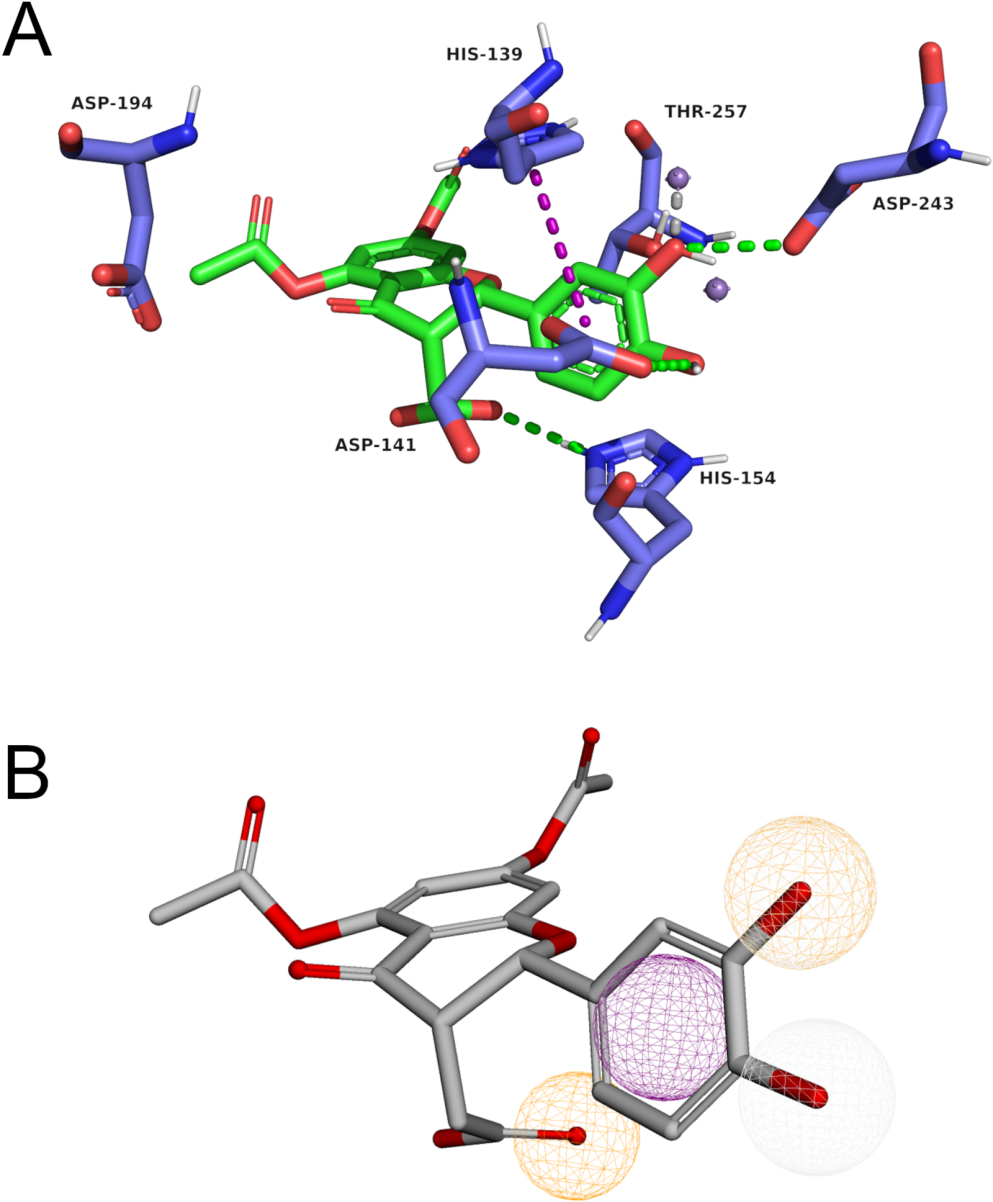
Molecular Docking and Pharmacophore map of Compound 18. (A) Predicted binding modes for compound 18 within the active site of *Lam*ARG. Compound 18 is depicted using green sticks, while the interacting residues of *Lam*ARG are represented by purple sticks, differentiated by atom type. Key interactions are highlighted in the figure, in which hydrogen bonds are indicated by green dotted lines, π‐π stacking interactions by purple dotted lines, cation‐π interactions by orange dotted lines, and ion‐dipole interactions by gray dotted lines. (B) Pharmacophore map constructed for *Lam*ARG based on the compound 18 and validated in the test set. Hydrogen bond acceptors are shown as yellow spheres, aromatic groups as purple spheres, and hydrogen bond donors as white spheres. The coordinates and sphere size for the pharmacophore map are detailed in Table 2.

The validated pharmacophore model developed for *Lam*ARG using compound 18 as the base, along with its corresponding predicted interaction profile, is presented in Figure 7.

### 3.5 Virtual Screening

#### 3.5.1 Pharmacophore‐Based Screening

The e‐Drug database plays a strategic role in drug repositioning studies due to its comprehensive repository of commercial drugs, offering detailed information on chemical structures, mechanisms of action, molecular targets, adverse effects, and drug interactions [60]. Integrating detailed data from the e‐Drug database with virtual screening (VS) tools facilitates an efficient and focused approach to discovering new treatments. This strategy, known for its cost‐efficiency, significantly reduces the time and expenses involved in drug development by streamlining the identification and prioritization of the most promising candidates for experimental testing [90].

After conducting pharmacophore screening in the e‐Drug database, 25 molecules were identified from the compound series exhibiting significant steric and electronic compatibility with the validated hypothesis. The alignment of these compounds with the pharmacophore requirements suggests their potential to modulate the *Lam*ARG receptor. Subsequently, these selected compounds were analyzed for their therapeutic applications in treating cutaneous leishmaniasis. Table 3 summarizes these findings by detailing the predicted binding affinities of the compounds, their structural alignments with the pharmacophore model, the various routes of administration, and prodrug status.

**Table 3:** Compounds selected from virtual screening by pharmacophore mapping.

Compound	Score (kcal/mol)	mRMSD (Å)	Route of administration	Prodrug
Arformoterol	−6.54	1.82	Inhalation	No
Dabigatran	−5.84	3.02	Oral	No
*R‐*Pirbuterol	−5.8	1.02	Inhalation	No
*SS*‐Formoterol	−5.52	4.5	Inhalation	No
*SR*‐Isoetharine	−5.5	1.76	Inhalation	Yes
*SR*‐Protokylol	−5.46	3.12	Oral	No
*S*‐Isoproterenol	−5.32	1.3	IV	No
*SS*‐Labetalol	−5.3	2.98	IV	No
Carbidopa	−5.02	2.16	Oral	No
Levalbuterol	−5	1.17	IV	No
*RR*‐Labetalol	−5	2.77	IV	No
Methyldopate	−4.91	2.2	IV	No
*SS*‐Isoetharine	−4.83	0.88	Inhalation	Yes
*S*‐Salmeterol	−4.75	5.42	Inhalation	No
Cefiderocol	−4.72	4.8	IV	No
Levodopa	−4.71	1.29	Oral	Yes
*R*‐Salmeterol	−4.68	5.16	Inhalation	No
*SR*‐Droxidopa	−4.49	5.56	IV	No
Methyldopa	−4.47	0.95	Oral	Yes
Levonordefrin	−4.4	1.1	IV	No
Epinephrine	−4.2	1.16	IV	No
Norepinephrine	−4.086	1.02	IV	No
Vilanterol	−3.81	2.76	Inhalation	No
Arbutamine	−3.28	1.77	IV	No
*SR*‐Labetalol	−0.88	2.02	IV	No

Among the potential inhibitors of *Lam*ARG identified through pharmacophore screening, Isoetharine, Levodopa, and Methyldopa were excluded from further consideration because they are classified as prodrugs. The rationale behind this is that prodrugs are derivatives or precursors of therapeutically active molecules, which are promptly converted into their active form through biotransformation in the body. As a result, the structure of the prodrug does not directly correspond to the molecular entity interacting with the therapeutic target [91]. This exclusion criterion ensures that only compounds with the appropriate structural characteristics for direct interaction with the pharmacological target are considered, thereby enhancing the likelihood of achieving the desired therapeutic effects.

The scores assigned to each compound in Table 3 indicate their predicted binding affinity to the pharmacological target, while the minimized root‐mean‐square deviation (mRMSD) values offer insights into their structural alignment with the pharmacophore. Lower mRMSD values suggest a closer correspondence to the pharmacophoric features, which may correlate with improved binding efficacy and increased activity against the intended target. This relationship is crucial for identifying compounds that not only demonstrate effective binding to their targets but also exhibit a favorable interaction profile for modulating biological receptors [56].

The pharmacophore screening results yield valuable insights into the binding affinities and structural alignments of the selected compounds within the validated pharmacophore model targeting the *Lam*ARG active site. Among the compounds evaluated, Arformoterol and Dabigatran exhibited the highest predicted binding affinities, with scores of −6.54 kcal/mol and −5.84 kcal/mol, respectively. Arformoterol, shows a relatively low minimized root mean square deviation (mRMSD) of 1.82 Å, suggesting a favorable structural alignment with the pharmacophore model. Dabigatran, despite having a slightly higher mRMSD of 3.02 Å, still presents a notable affinity score (−5.84 kcal/mol), indicating that it remains favorable for potential repositioning applications [56].

*R‐*Pirbuterol also shows promise based on the pharmacophore search, achieving a binding affinity score of −5.8 kcal/mol and a notably low mRMSD of 1.02 Å, thereby indicating a favorable structural match to the pharmacophore. Similarly, *SR‐*Protokylol, with a binding affinity of −5.46 kcal/mol and an mRMSD of 3.12 Å, exhibits a reasonable alignment with the pharmacophore model, though not as optimal as *R‐*Pirbuterol. Notably, other β‐adrenergic agonists, such as *S‐*Isoproterenol and Levalbuterol, also exhibit favorable binding affinities and compatibility with the pharmacophore model (Table 3). Conversely, compounds exhibiting higher mRMSD values, such as Salmeterol (R and S isomers) and *SS‐*Formoterol (Table 3), are likely to present a suboptimal interaction profile with *Lam*ARG [56].

Interestingly, Cefiderocol, a recently approved siderophore cephalosporin antibiotic with proven efficacy against multidrug‐resistant pathogens [92], was also identified during the pharmacophore screening. The screening results revealed that Cefiderocol exhibited an affinity score of −4.72 kcal/mol, alongside a relatively high RMSD value of 4.8 Å. This suggests potentially suboptimal interaction with the target receptor.

Our findings therefore indicate that Dabigatran and β‐adrenergic agonists, including Arformoterol, and Protokylol, could potentially interact with key elements of the *Lam*ARG binding site, which is determinant for their prospective efficacy as therapeutic agents against cutaneous leishmaniasis. The favorable binding affinities, combined with their close alignment with the pharmacophore, provide a rationale for further investigation into these compounds as candidates for drug repositioning efforts [93].

#### 3.5.2 Docking of Potential *Lam*ARG Inhibitors

Following the virtual screening within the E‐Drug database, we proceeded with molecular docking simulations to elucidate the binding modes established between the potential *Lam*ARG inhibitors at the active site. While pharmacophore modeling provides preliminary insights into ligand characteristics, it is limited in detailing the specific interactions that govern protein‐ligand recognition [81]. In this regard, molecular docking provides valuable insights into binding modes and affinities, enabling visualization of potential inhibitors' interactions at the atomic level. Therefore, this technique is capable of elucidating key interaction characteristics such as hydrogen bonding, π‐π stacking, and steric factors, which determine binding efficacy. This approach proven valuable to refine candidate selection and prioritize compounds for further experimental validation [23, 94, 95].

Thus, the drugs selected through pharmacophore screening, underwent characterization of their binding modes and affinities to the *Lam*ARG active site using molecular docking analysis, as shown in Table 4. Ionic interactions were evaluated but not included in Table 4, as none were identified in the docking results.

**Table 4:** Docking affinity scores and predicted binding modes for potential inhibitors of *Lam*ARG interacting with the target protein.

Drug	Score	Hydrogen bond	π‐π	Cation‐π	Ion‐dipole	Salt bridge
Carbidopa	38.34	D243, D141, N143	—	—	Mn^+2^	—
Vilanterol	34.00	N143, D243	H154	H154	Mn^+2^	—
*S‐*Salmeterol	33.97	H154, D243, D245	H139	H139	Mn^+2^	—
*R*‐Salmeterol	33.74	N143, D243	H28	—	—	—
Levalbuterol	30.66	D137, H154	—	—	Mn^+2^	—
*R*‐Pirbuterol	29.12	D243, H154	H154	H139	—	—
Dabigatran	28.45	N152, H154, E288	H139	—	—	—
*RR*‐Labetalol	28.13	D141, A192, E197	H139	H139	Mn^+2^	—
Arbutamine	28.02	N143, D243	H139	H139	Mn^+2^	—
Epinephrine	28.00	D141, H154, D243	H154	—	Mn^+2^	—
*SS*‐Formoterol	27.81	H154	—	—	Mn^+2^	c
Methyldopate	27.67	H139, D141, D243	H139	H154	Mn^+2^	—
Norepinephrine	27.29	D141, D243	H139	—	Mn^+2^	—
*SR*‐Droxidopa	27.27	N152, H154, D243	—	—	Mn^+2^	H154
Levonordefrin	26.92	D141, N152, H154, D243	—	—	Mn^+2^	H154
*SR*‐Protokylol	26.44	D141, H154, E197, D243, D245	H139	H154	Mn^+2^	—
*RR*‐Arformoterol	25.76	T257, H154	—	—	Mn^+2^	—
*S*‐Isoproterenol	24.95	D243, D141	H139	—	—	—
*SS*‐Labetalol	24.68	D243, E197, D141, N143, T257	H154	—	—	—
*SR*‐Labetalol	23.75	H154	—	—	—	—
Cefiderocol	21.31	N152, D245	H139	—	—	H154

**Figure 8:**
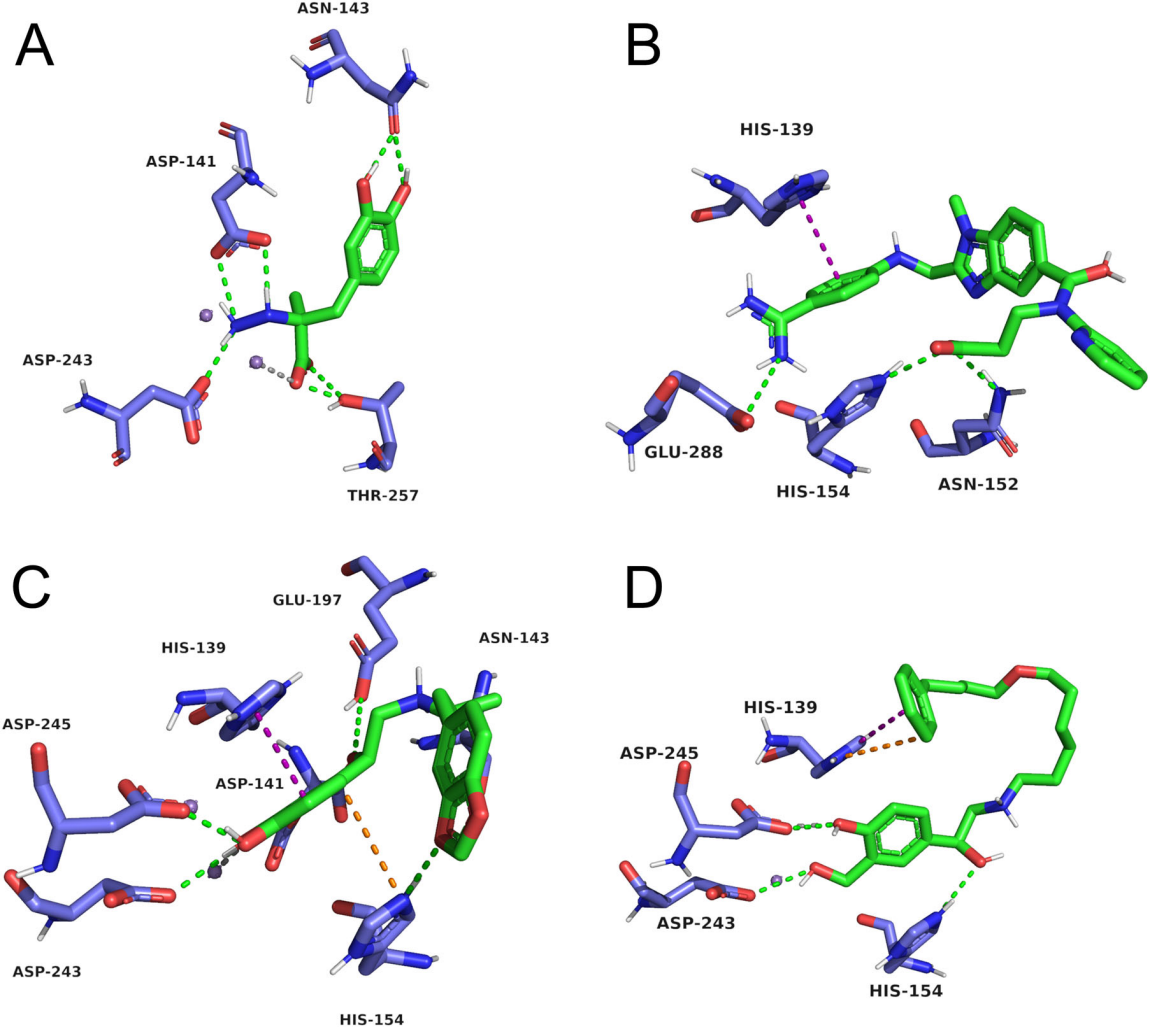
Predicted binding modes for potential inhibitors of *Lam*ARG obtained through molecular docking simulations. (A) Carbidopa. (B) Dabigatran. (C) *SR‐*Protokylol. (D) *S‐*Salmeterol. The ligand is depicted in green, while the interacting residues of *Lam*ARG are represented in purple. Hydrogen bonds are illustrated with green dotted lines, π‐π stacking interactions with purple dotted lines, cation‐π interactions with orange dotted lines, and ion‐dipole interactions with gray dotted lines.

Among the tested compounds (Table 4), Carbidopa emerges as the top‐scoring ligand with a docking score of 38.34, thus indicating a particularly stable interaction within the *Lam*ARG binding site. Furthermore, it exhibits a promising interaction profile with the target by forming hydrogen bonds with the catalytic triad residues Asp243 and Asp141, while also establishing an ion‐dipole interaction with the Mn²⁺ cofactor ion (Figure 8A) [24]. These interactions likely contribute to Carbidopa's high affinity, as the presence of negatively charged residues such as aspartates and the metal ion coordination can create strong electrostatic attractions, stabilizing the protein‐ligand complex.

Notably, Dabigatran, a high‐scoring ligand, exhibited a distinct interaction pattern among the tested inhibitors by engaging with both Glu288 and Asp154—key residues for substrate binding in *Lam*ARG. Dabigatran also established a π‐π stacking interaction with His139, which functions as a proton carrier within the catalytic triad [23]. This interaction profile (Figure 8B) suggests that Dabigatran may effectively interfere with catalytic alignment, potentially competing with the natural substrate at the active site, thereby hindering catalytic progression.

*SR‐*Protokylol and *S‐*Salmeterol also demonstrated a particularly consistent interaction network (Figure 8C, D), binding not only with His154 but also with all metal‐coordinating residues within the *Lam*ARG active site (i.e., His139, Asp243, and Asp245), along with the Mn²⁺ cofactor. Noteworthy, Protokylol also formed a hydrogen bond with Asp141, a component of the enzyme's catalytic triad [23]. This extensive network of interactions suggests that these compounds could effectively disrupt metal coordination and catalytic alignment, thereby reinforcing their potential as *Lam*ARG inhibitors [24].

At the lower end of the scoring spectrum, Cefiderocol (21.31) and *SR‐*Labetalol (23.75) demonstrate limited interactions with the target enzyme (Figure S4). Cefiderocol's binding is predominantly confined to Asn152 and Asp245, forming an additional π‐π interaction with His139. This suggests that it may not attain optimal stabilization within the binding pocket. Likewise, *SR‐*Labetalol primarily engages with His154 and His139 but lacks a robust hydrogen bond network, which likely contributes to its lower docking score.

Overall, our findings presented in Table 4 indicate that particularly Carbidopa, *S‐*Salmeterol, Dabigatran, and *SR*‐Protokylol exhibit an interaction profile that further supports their potential as *Lam*ARG inhibitors. This potential is primarily attributed to the predicted binding affinities of these compounds, which are comparable to those of established *Lam*ARG inhibitors (Table 1), as well as their interactions with residues directly involved in the enzyme's catalytic mechanism. Among them, these interactions involve residues from the catalytic triad (i.e., Glu288, Asp141, and His139), substrate‐binding (i.e., His154 and Glu288), and metal cofactor coordination (i.e., His139, Asp243, and Asp245) [24]. Notably, all other screened compounds also established interactions with at least one key residue of the *Lam*ARG active site (Figures S4 to S5), albeit with reduced binding affinities or fewer key interactions.

It is worth noting that the docking scores of these four selected drugs fall within or even exceed the range observed for bioactive compounds with experimentally reported activity against *Lam*ARG, as presented in Table 1. In this series, catechin exhibited the highest predicted binding score, at 29.69, whereas compound 20 showed the lowest, at 15.17. Notably, two of the selected drugs, Carbidopa and *S*‐Salmeterol, demonstrated docking scores higher than the maximum observed among the bioactive compounds, while the other two, *SR‐*Protokylol and Dabigatran, also fall within the range of these values. This comparison further reinforces the potential relevance of these compounds as promising candidates in the context of *Lam*ARG inhibition. As a result, these compounds may still act as potential *Lam*ARG inhibitors, but they are less likely candidates.

#### 3.5.3 Post‐Processing Analysis

Although Arformoterol, *R*‐Pirbuterol, *SS‐*Formoterol, *R*‐Salmeterol, *S*‐Salmeterol, and Vilanterol were identified as potential *Lam*ARG modulators through pharmacophore screening, their formulations as inhalable medications considerably limit their practical application in treating systemic conditions such as leishmaniasis, as they are primarily designed to target the pulmonary region [95]. This exclusion ensures a more focused approach in identifying potential therapies that can effectively address the specific pathophysiology of leishmaniasis.

Consequently, among the potential *Lam*ARG modulators identified through pharmacophore screening on E‐Drug, only Protokylol and Dabigatran were selected for further evaluation, as they meet the criteria for oral administration, which is particularly advantageous for enhancing patient adherence to treatment regimens. Using the range defined by catechin and compound 20, bioactive molecules experimentally validated as positive controls in this study (Table 1), it is evident that the binding affinities of Protokylol (26.44) and Dabigatran (28.45) also fall within this interval. Catechin exhibited the highest predicted binding affinity among the experimental controls, at 29.69, while compound 20 had the lowest at 15.17. The inclusion of these experimentally validated compounds as positive controls provides a valuable benchmark for comparison and helps contextualize the binding affinities observed in our study. Notably, the alignment of Protokylol and Dabigatran within this established range further corroborates the validity of our docking results and reinforces their potential as promising *Lam*ARG inhibitors identified through pharmacophore‐based virtual screening.

While intravenous drugs like Isoproterenol can effectively achieve systemic targeting, they often pose significant obstacles in terms of patient compliance and practical application, including issues related to accessibility, discomfort, and the need for clinical supervision during administration [94]. Thus, prioritizing orally administered medications ensures a more practical and effective approach to treating leishmaniasis while optimizing patient adherence to therapy.

Although Carbidopa is primarily administered orally, it was excluded from further evaluation due to its common co‐formulation with Levodopa. Carbidopa acts by inhibiting aromatic l‐amino acid decarboxylase (AADC) enzymes in peripheral tissues, thereby preventing the premature conversion of Levodopa into dopamine outside the central nervous system. This inhibition significantly increases the amount of Levodopa that crosses the blood‐brain barrier, where it is subsequently converted into dopamine, effectively managing the motor symptoms associated with Parkinson's disease [96]. Nevertheless, this dependence on coadministration restricts its applicability for direct drug repositioning, particularly for conditions that do not benefit from this synergistic effect. The complex pharmacokinetics and narrow therapeutic context of this combination make it less suitable for broader repurposing strategies.

The selected compounds exhibit markedly distinct therapeutic indications, targeting unique physiological systems. Protokylol acts as a beta‐adrenergic agonist, mainly indicated as a bronchodilator for respiratory conditions like asthma and chronic obstructive pulmonary disease (COPD). Its mechanism involves relaxing smooth muscles in the airways to enhance airflow and relieve symptoms associated with these conditions. Meanwhile, Dabigatran is used in managing thromboembolic disorders, including stroke prevention in patients with atrial fibrillation, as well as in the treatment and prevention of deep vein thrombosis (DVT) and pulmonary embolism (PE). It exerts its effects by inhibiting thrombin, which prevents the formation of blood clots and reduces the risk of complications associated with clotting [97].

Given their established therapeutic usage and well‐documented safety profiles [98, 99], in addition to a significant pharmacophoric correspondence (Table 3) and favorable binding modes (Figure 8), Protokylol and Dabigatran hold promise for drug repositioning in the treatment of cutaneous leishmaniasis. This integrated approach leverages existing safety data to accelerate new treatment development, effectively addressing the urgent need for effective therapies in this field [100]. By focusing on these compounds, researchers can streamline the clinical evaluation process, leading to quicker access to innovative solutions and enhancing the likelihood of successful outcomes [36] while advancing research in neglected tropical diseases.

### 3.6 Molecular Dynamics Simulations

In virtual screening campaigns, docking and molecular dynamics (MD) simulations form a complementary workflow to evaluate potential drug candidates. While docking serves as a preliminary step in virtual screening by generating static protein‐ligand complexes and providing initial estimates of binding affinities, it has significant limitations in capturing the conformational flexibility inherent in biological systems and the involvement of solvent molecules in these interactions. To address these shortcomings, MD simulations are often employed after docking to corroborate and refine these preliminary findings [101].

By accurately reproducing the dynamic behavior of proteins and ligands under physiological conditions, MD simulations provide deeper insights into the stability of binding poses, alternative binding modes, and the influence of solvent molecules in the interaction. Additionally, when integrated with methods like MM‐GBSA, MD simulations can yield even more precise estimates of binding affinities, which is invaluable for evaluating the effectiveness of potential drug candidates [67]. This capability allows researchers to assess whether selected compounds are likely to remain stable in the active site and be suitable for subsequent experimental evaluation [101], ultimately leading to a more informed and directed drug repositioning process [102].

To evaluate the equilibrium of the molecular dynamics’ simulation performed for the *Lam*ARG target, we analyzed the RMSD, Rg, and SASA of the simulated complexes, i.e., *Lam*ARG‐Dabigatran and *Lam*ARG‐Protokylol. RMSD assesses the average deviation in atomic positions relative to the initial simulation structure, providing insights into conformational fluctuations over time. Similarly, Rg evaluates the degree of structural compactness, indicating changes in the spatial arrangement of atoms relative to the structure's center of mass. Lastly, SASA estimates the solvent‐exposed surface area of the complex, reflecting variations in the accessibility of protein residues [103].

**Figure 9:**
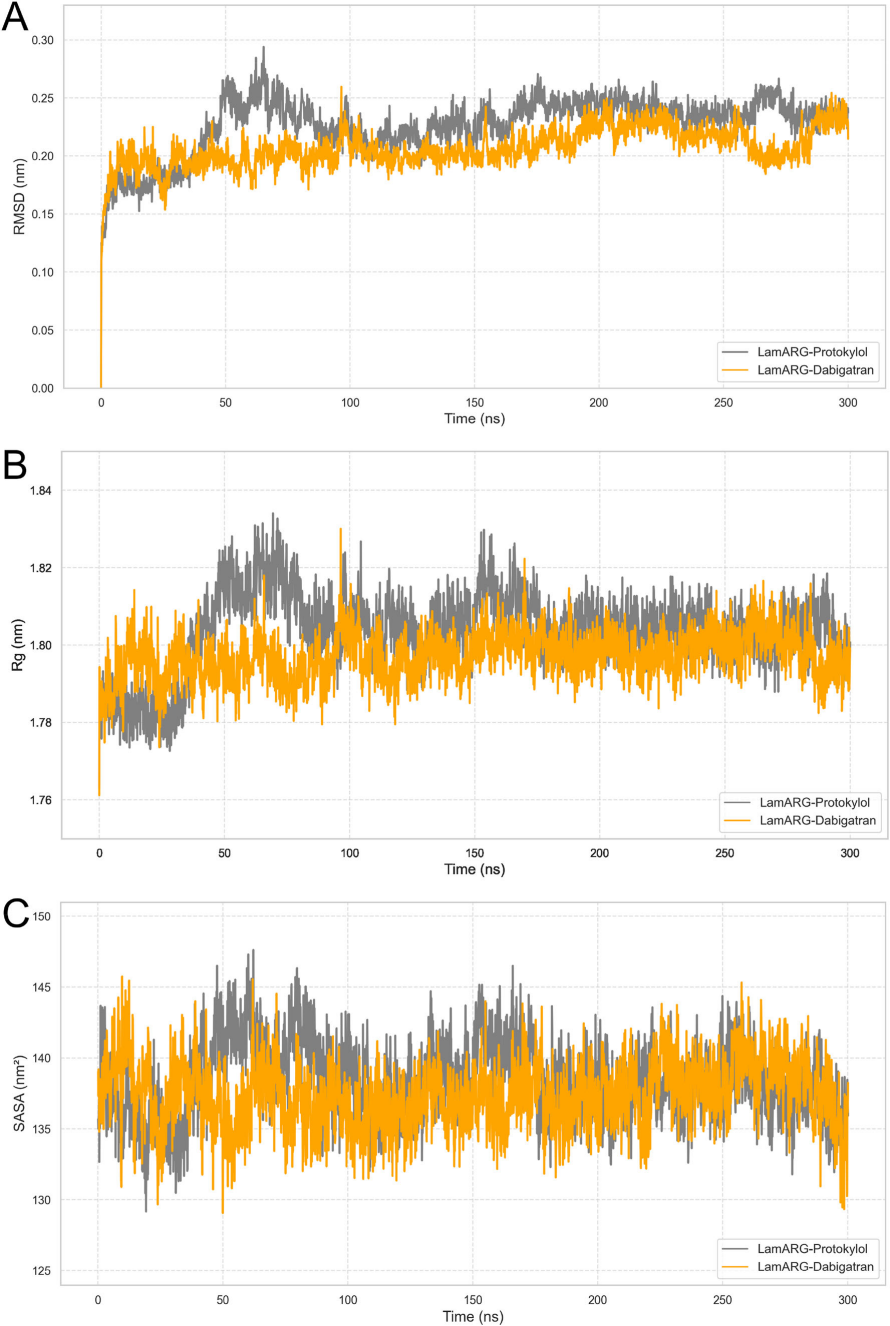
RMSD, Rg, and SASA analyses of *Lam*ARG. (A) RMSD values calculated from the *Lam*ARG backbone atoms are shown as a function of time. (B) Rg values calculated from the backbone atoms are shown as a function of time. (C) SASA values calculated from the protein atoms are shown as a function of time.

Regarding the RMSD analysis (Figure 9A), the initial effects of the simulation—specifically those observed during the first 150 ns—were excluded from subsequent comparisons to ensure meaningful analysis. This decision is supported by the observation of an initial period of structural instability in all trajectories. This instability may be attributed to the kinetic shock experienced by the molecular systems during the early stages of the simulation process [104]. Consequently, the variations in structural parameters during this period were not representative of the protein's behavior. Following this phase, RMSD values reached stable plateaus for *Lam*ARG, which persisted until the end of all trajectories, indicating that the protein structures fluctuated around average stable conformations [105]. Similarly, Rg and SASA values also stabilized at plateaus after this period in the analyzed simulations, indicating that all studied systems achieved dynamic equilibrium during the latter half of the trajectories. This behavior suggests that the structural parameters observed during this period are representative of the molecular dynamics of the analyzed complexes [67].

**Figure 10:**
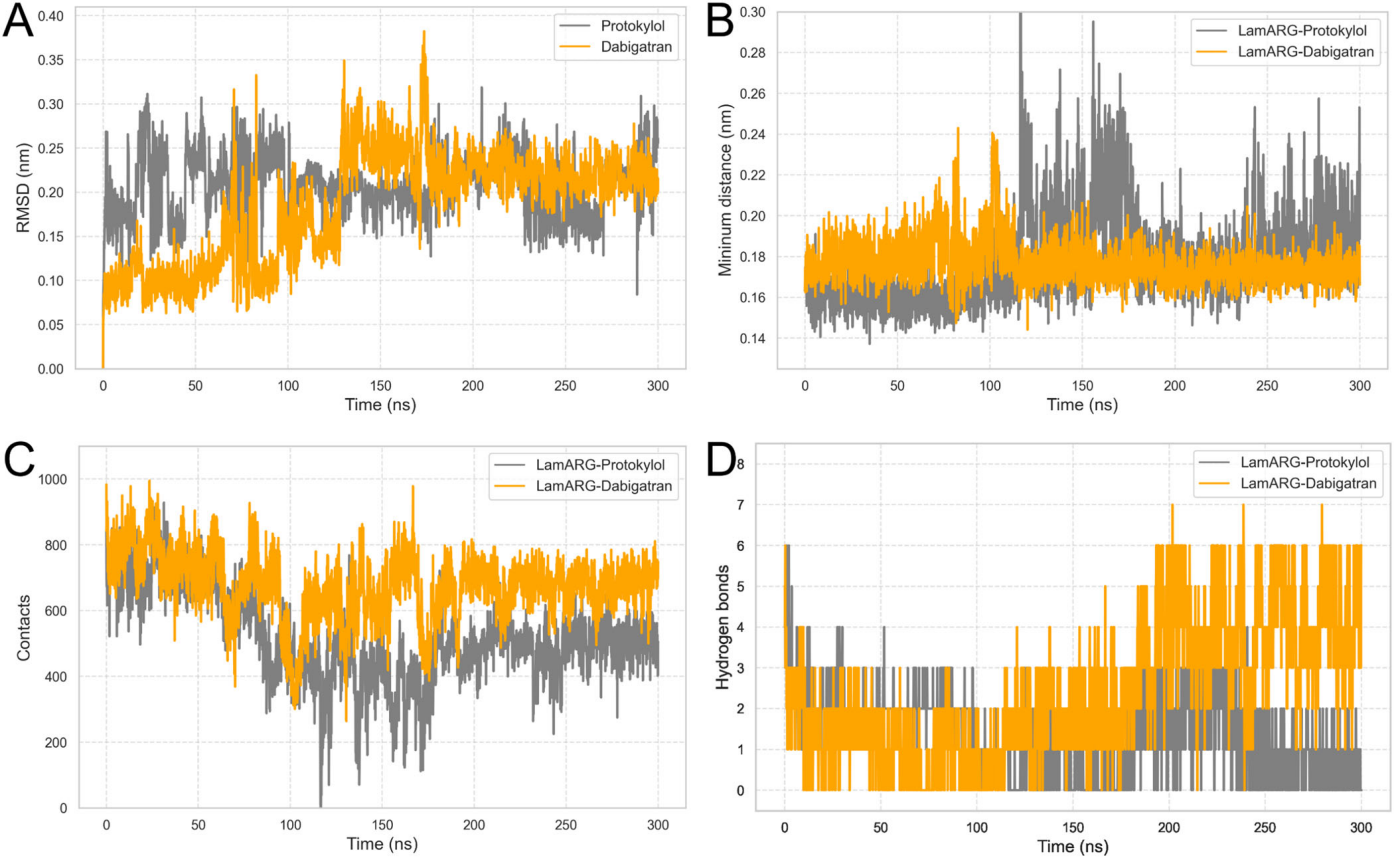
Analyses of ligand RMSD, minimum distances, number of contacts and binding energy. (A) Ligand RMSD, showing the RMSD of ligand atoms relative to the initial structure over time. (B) Minimum distances computed between the ligand and the *Lam*ARG binding site during the simulation. (C) Number of contacts formed between the ligand and the protein over the simulation. (D) Binding energy estimated for the complexes over time, calculated using the MM‐GBSA method.

To assess complex stability, we initially computed the RMSD of the ligands Protokylol and Dabigatran individually, as shown in Figure 10A. This analysis reveals that Dabigatran exhibited stable behavior when complexed with *Lam*ARG, with its RMSD values stabilizing around consistent plateaus from 150 ns onward across the simulation. These results suggest that the ligand maintained stable binding throughout the simulation [67]. Protokylol, on the other hand, exhibited relatively unstable behavior throughout the simulation, including during the system equilibration period. Its RMSD values showed significant fluctuations, reflecting a lack of consistent binding to the *Lam*ARG active site.

The minimum distances between *Lam*ARG and the ligands, as shown in Figure 10B, revealed distinct behaviors for the Protokylol‐*Lam*ARG and Dabigatran‐*Lam*ARG complexes during the simulations. In particular, the Protokylol‐*Lam*ARG complex exhibited significant fluctuations in minimum distances, especially during the interval between 120 and 170 ns. As depicted in Figure S6, this instability can be attributed to Protokylol shifting out of the enzyme's active site, reducing its ability to maintain consistent interactions. In contrast, Dabigatran remained firmly associated with *Lam*ARG's active site throughout the simulation, displaying relatively lower and more stable minimum distance values (0.17 ± 0.01 nm) compared to Protokylol (0.19 ± 0.02 nm). These findings underscore the greater stability of the Dabigatran‐*Lam*ARG complex, further supporting Dabigatran's potential as a robust inhibitor candidate.

The number of contacts within 5 Å was also analyzed (Figure 10C), serving as an indicator of potential interactions between the protein and ligand groups [106]. As shown in Figure 10C, the Protokylol‐*Lam*ARG complex exhibited a significant reduction in the number of contacts over time, particularly between 120 and 170 ns. As previously discussed, this behavior corresponds to the period during which Protokylol moved out of the *Lam*ARG active site (Figure S6). As a result, the number of molecular interactions was substantially reduced (470.87 ± 87.54), compromising the stability of the complex. In contrast, the Dabigatran‐*Lam*ARG complex maintained a relatively constant and notably higher number of contacts throughout the simulation (674.83 ± 76.33), particularly after system equilibration (i.e., during the second half of the simulation). This analysis, in conjunction with the minimum distance results, reveals that Dabigatran remained at a relatively short and constant distance from *Lam*ARG throughout the entire simulation, indicating a stable and sustained interaction profile with the enzyme.

To further monitor the complex interactions throughout the simulation, we analyzed the number of hydrogen bonds formed over time, as hydrogen bonding is a key interaction type in molecular recognition [107]. Overall, the Protokylol‐*Lam*ARG complex exhibited a slightly lower number of hydrogen bonds throughout most of the simulation, with an average of 1.16 ± 0.96, compared to the Dabigatran‐*Lam*ARG complex, which formed an average of 3.57 ± 1.43 hydrogen bonds. Conversely, the higher number of hydrogen bonds in the Dabigatran‐*Lam*ARG complex points to a stronger and more sustained interaction profile, which is likely central to keeping Dabigatran firmly anchored within the active site, thereby ensuring complex stability.

A hydrogen bond occupancy analysis was additionally performed to gain a deeper understanding of how hydrogen bonds are formed. Hydrogen bond occupancy is defined as the percentage of total conformations recorded throughout the simulation in which a ligand establishes at least one interaction of this type with a protein residue [103]. In the case of Dabigatran, it consistently interacted with residues of *Lam*ARG's active site, including Asp141 (88.61%), Asn152 (73.68%), and Thr257 (61.03%). Among these, Asp141 is a component of the catalytic triad of *Lam*ARG [23], so that the consistent interaction between Dabigatran and this residue further suggests that it may acts as a potential inhibitor of *Lam*ARG, supporting its candidacy for drug repositioning. Additionally, the high hydrogen bond occupancy with Asn152 and Thr257 highlights the importance of these residues in maintaining the complex's stability. Consequently, the interaction profile computed throughout the MD simulations supports the notion that Dabigatran forms a relatively stable and favorable interaction profile with *Lam*ARG.

Protokylol, in turn, displayed distinct behavior during the simulation. Hydrogen bond occupancy primarily involved residues outside the active site, i.e., Leu293 (26.78%) and Pro249 (21.12%), with notably limited occupancy values. This aligns with the reduced average number of hydrogen bonds formed during the simulation of *Lam*ARG‐Protokylol, as previously observed. Additionally, this discrepant interaction profile, in contrast to the docking predictions, can be attributed to the snapshot nature of docking analyses, which capture a single moment of interaction. In docking, the selected pose typically represents a highly favorable energy state at a given instant [67]. Nonetheless, the simulation over time revealed that Protokylol was unable to maintain stable binding within the active site, ultimately shifting out of it.

Finally, to gain deeper insights into the stability of the complexes, we estimated the binding energy for *Lam*ARG‐Protokylol and *Lam*ARG‐Dabigatran using an MM‐GBSA analysis, as shown in Figure 10D. Binding affinity reflects the change in free energy associated with the formation of a molecular complex. This parameter is fundamental for assessing the strength of protein‐ligand interactions and serves as a key indicator of a compound's pharmacological potential (Changhao [108]). Since binding free energy is computed by subtracting the energy of the individual components (i.e., the protein and ligand) from the energy of their complexed state, negative free energy values indicate spontaneous and energetically favorable interactions, whereas positive values suggest unfavorable processes [109].

Dabigatran exhibited a numerically lower binding energy with *Lam*ARG (−108.96 ± 32.72 kJ/mol), indicating a more favorable interaction compared to Protokylol (−63.73 ± 18.46 kJ/mol). This outcome aligns with previous MD findings, where hydrogen bond analysis revealed consistent and strong interactions with residues from the active site. These interactions were accompanied by a lower minimum distance and a higher number of contacts over time, further supporting the favorable binding behavior observed for Dabigatran. Conversely, Protokylol displayed considerably higher binding energy, suggesting less favorable binding, as reflected by the reduced number of hydrogen bonds and fewer contacts formed over time.

Our findings demonstrate that Dabigatran exhibits superior stability and favorable binding modes compared to Protokylol, consistently interacting with key residues in the enzyme's active site, including Asp141, a component of the catalytic triad [23]. These results position Dabigatran as a promising candidate for drug repurposing, requiring further experimental validation to confirm its potential.

## 4 Conclusions

This study provided a validated theoretical model of the *Lam*ARG protein. The compilation of known inhibitors of *Lam*ARG from the literature and databases yielded 20 recognized enzyme inhibitors, from which 995 corresponding decoys were generated. Molecular docking results revealed key interactions between these inhibitors and the *Lam*ARG active site, which provided the basis for constructing individual pharmacophore hypotheses. Among these, pharmacophore hypothesis number 18 demonstrated satisfactory predictive performance during the validation process, in which the model was challenged using a test set composed of known *Lam*ARG inhibitors and their corresponding decoys.

The validated pharmacophore was used as a reference for virtual screening in the e‐Drug database, which identified 22 compounds with significant steric‐electronic alignment to the validated pharmacophore map, thus indicating their potential to inhibit the *Lam*ARG enzyme. Among these compounds, Protokylol and Dabigatran were selected for subsequent MD simulations because they are preferably administered orally and are available in their active, non‐metabolized forms in commercial formulations.

Molecular docking and dynamics further reinforce Dabigatran as a promising *Lam*ARG inhibitor, consistently interacting with residues from the enzyme's active site, including Asp141, a component of the catalytic triad. In contrast, Protokylol exhibited unfavorable binding modes over time, rendering it unsuitable as a viable inhibitor.

Our findings, therefore, suggest that Dabigatran holds potential as an effective agent for treating cutaneous leishmaniasis. This compound offers opportunities to improve the quality of life for individuals affected by or at risk of this disease. Nonetheless, additional experimental assays are necessary to validate the efficacy of Dabigatran and confirm its repositioning potential [29]. Upon successful validation, there is a significant opportunity to develop a more targeted and less toxic treatment, which is determinant in ensuring treatment efficacy and adherence [110].

## Supporting information


**Supporting Figure S1:** Predicted binding modes for known *Lam*ARG inhibitors (Part 1). **Supporting Figure S2:** Predicted binding modes for known *LamARG* inhibitors (Part 2). **Supporting Figure S3:** Predicted binding modes for known *Lam*ARG inhibitors (Part 3). **Supporting Figure S4:** Predicted binding modes for potential *Lam*ARG inhibitors (Part 1). **Supporting Figure S5:** Predicted binding modes for potential *Lam*ARG inhibitors (Part 2). **Supporting Figure S6:** Three‐dimensional structure of the *Lam*ARG‐Protokylol complex before and after molecular dynamics (MD) simulations.

## Data Availability

The data that supports the findings of this study are available in the Supporting material of this article.
