# Supplementary material for: Unveiling Novel Arginase Inhibitors for Cutaneous Leishmaniasis Using Drug Repurposing and Virtual Screening Approaches
Source: J Cell Biochem. 2025 Aug 22;126(8):e70060. doi: 10.1002/jcb.70060 (PMC12374090; doi:10.1002/jcb.70060)
Supplement: Supplementary file 1 — Supporting Figure S1: Predicted binding modes for known LamARG inhibitors (Part 1). Supporting Figure S2: Predicted binding modes for known LamARG inhibitors (Part 2). Supporting Figure S3: Predicted binding modes for known LamARG inhibitors (Part 3). Supporting Figure S4: Predicted binding modes for potential LamARG inhibitors (Part 1). Supporting Figure S5: Predicted binding modes for potential LamARG inhibitors (Part 2). Supporting Figure S6: Three‐dimensional structure of the LamARG‐Protokylol complex before and after molecular dynamics (MD) simulations. [file JCB-126-e70060-s001.docx]

SUPPORTING INFORMATION


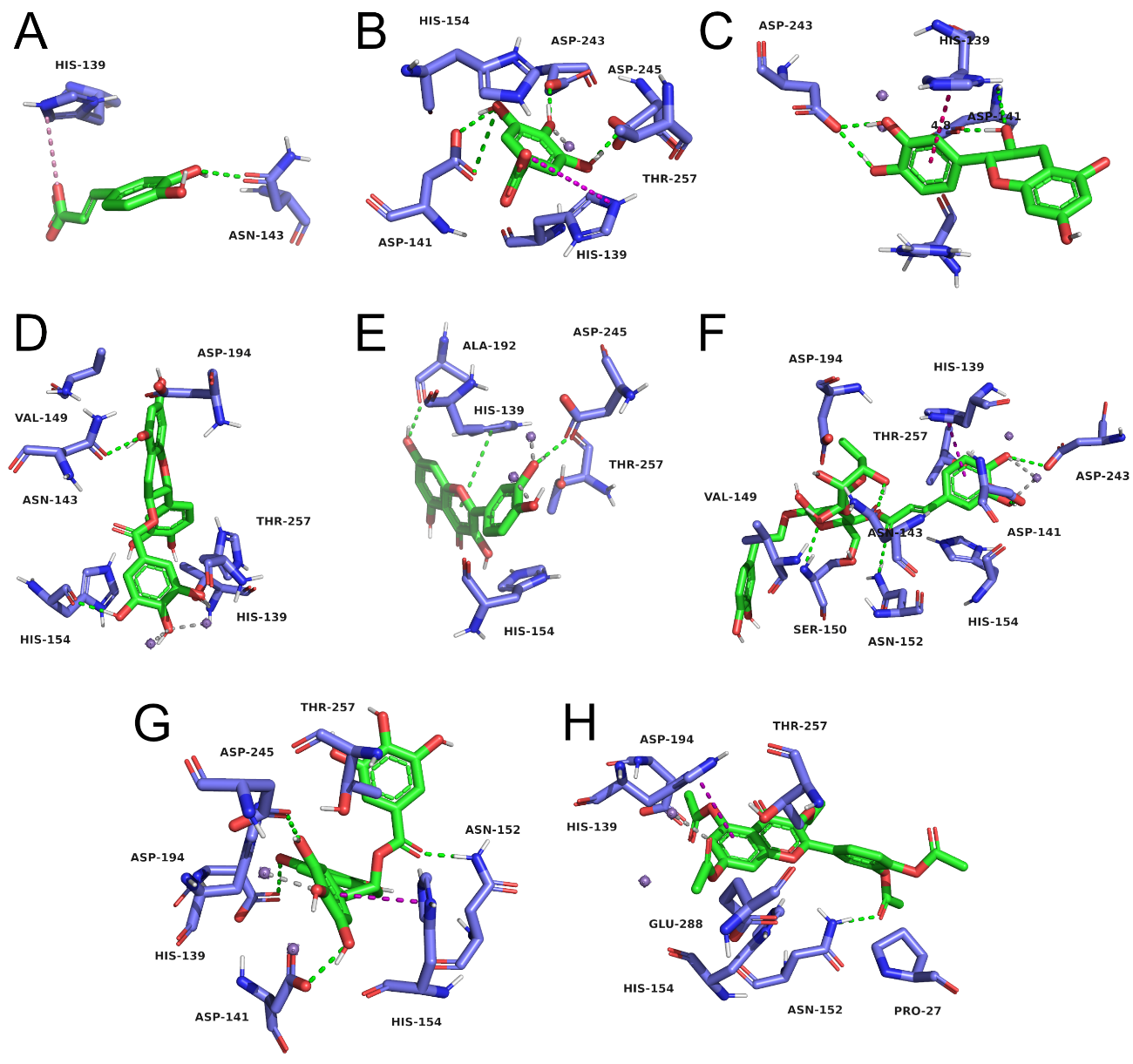


**Figure S1** **Predicted binding modes for known *Lam*ARG inhibitors (Part 1).** (A) Caffeic acid. (B) Gallic acid. (C) Catechin. (D) Epigallocatechin-3-Gallate. (E) Quercetin. (F) Compound 6. (G) Compound 7. (H) Compound 8. The ligand is depicted in green, while the interacting residues of *Lam*ARG are represented in purple. Hydrogen bonds are illustrated with green dotted lines, π-π stacking interactions with purple dotted lines, cation-π interactions with orange dotted lines, and ion-dipole interactions with gray dotted lines. Residues interacting through hydrophobic interactions with the inhibitors are also displayed in the figure.

**
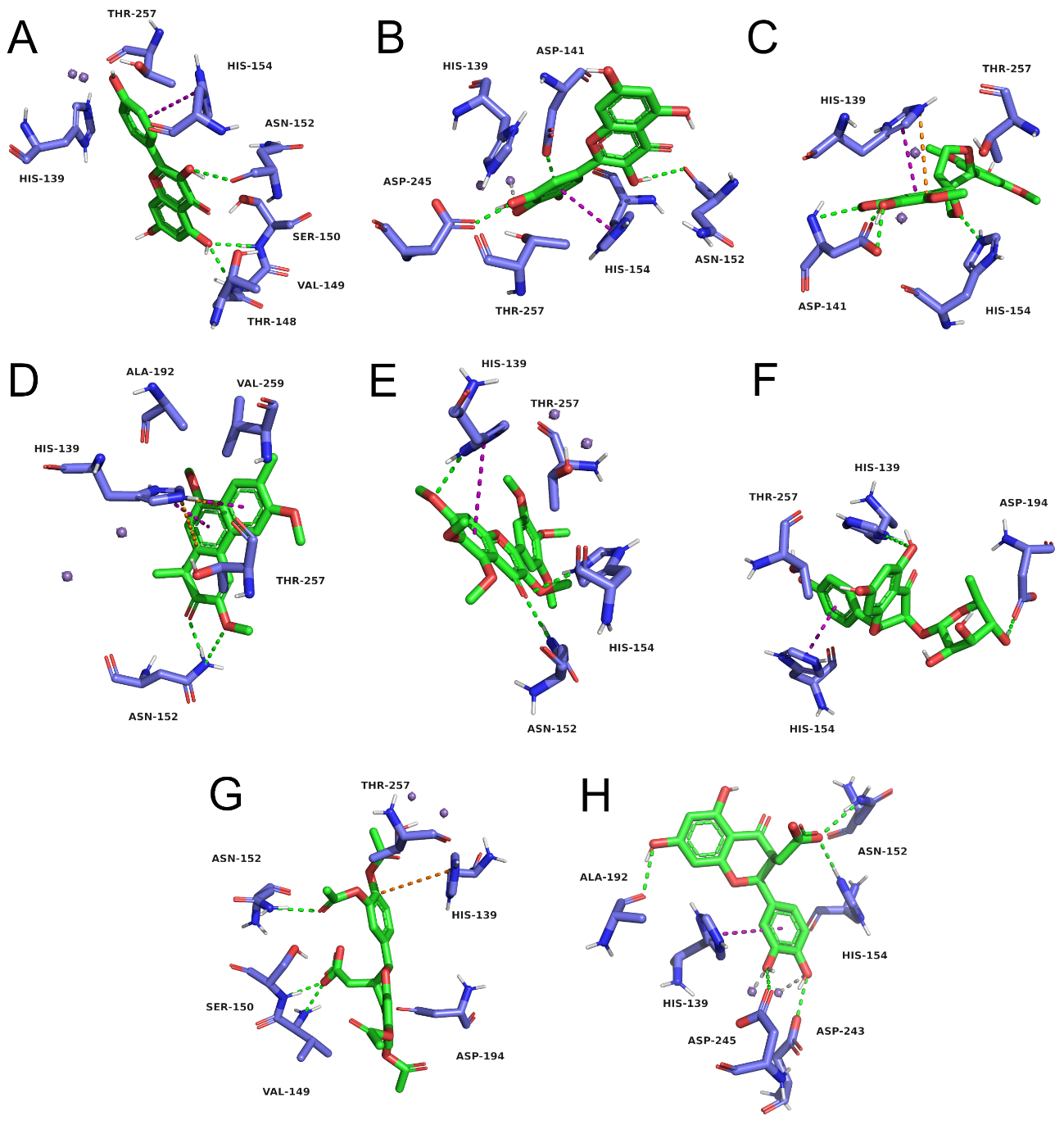
Figure S2** **Predicted binding modes for known *LamARG* inhibitors (Part 2).** (A) Compound 9. (B) Compound 10. (C) Compound 11. (D) Compound 12. (E) Compound 13. (F) Compound 14. (G) Compound 15. (H) Compound 16. The ligand is depicted in green, while the interacting residues of *Lam*ARG are represented in purple. Hydrogen bonds are illustrated with green dotted lines, π-π stacking interactions with purple dotted lines, cation-π interactions with orange dotted lines, and ion-dipole interactions with gray dotted lines. Residues interacting through hydrophobic interactions with the inhibitors are also displayed in the figure.


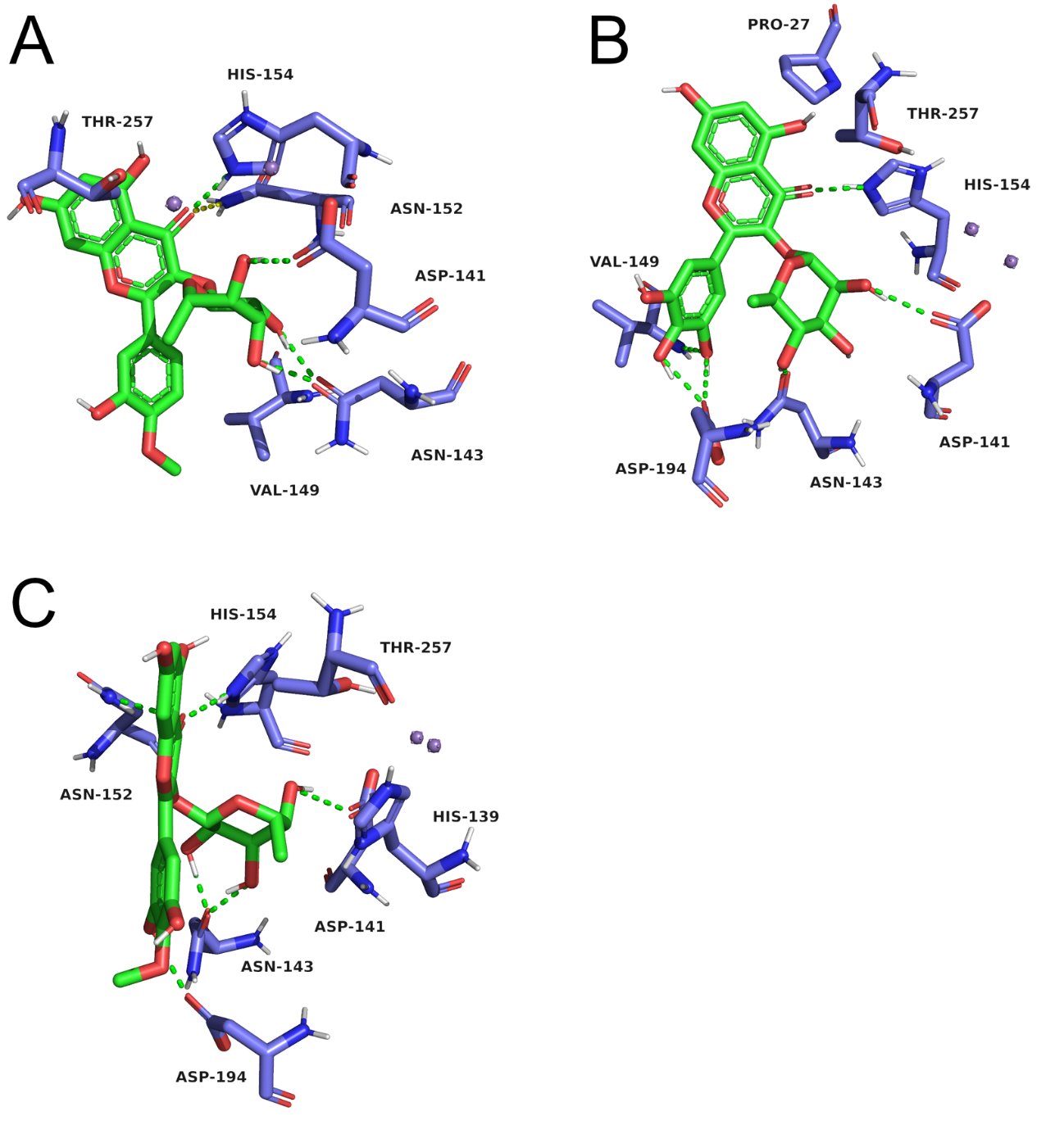


**Figure S3** **Predicted binding modes for known *Lam*ARG inhibitors (Part 3).** (A) Compound 17. (B) Compound 19. (C) Compound 20. The ligand is depicted in green, while the interacting residues of *Lam*ARG are represented in purple. Hydrogen bonds are illustrated with green dotted lines, π-π stacking interactions with purple dotted lines, cation-π interactions with orange dotted lines, and ion-dipole interactions with gray dotted lines. Residues interacting through hydrophobic interactions with the inhibitors are also displayed in the figure.

**
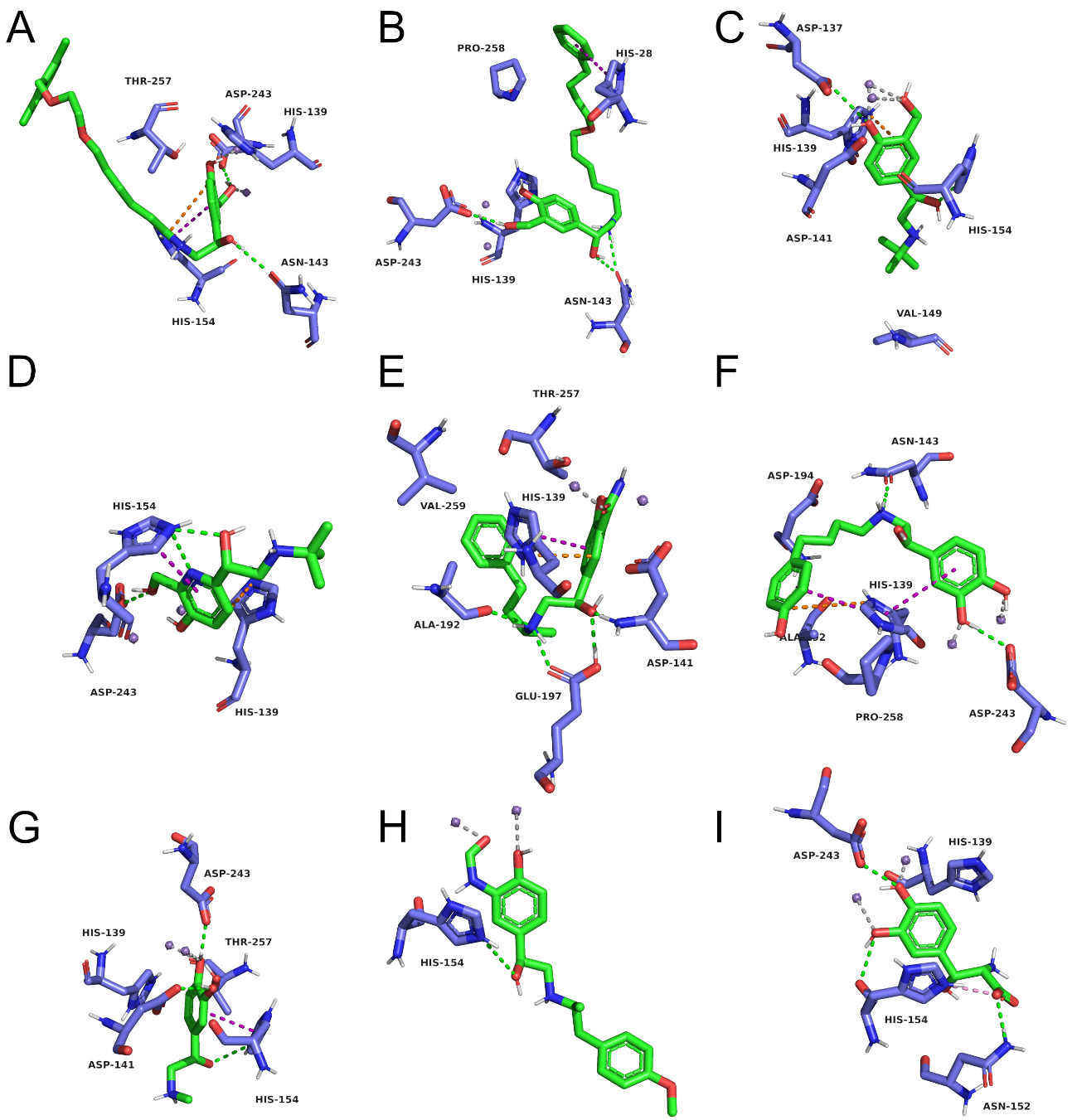
Figure S4** **Predicted binding modes for potential *Lam*ARG inhibitors (Part 1).** (A) Vilanterol. (B) *R*-Salmeterol. (C) Levalbuterol. (D) *R*-Pirbuterol. (E) *RR*-Labetalol. (F) Arbutamine . (G) Epinephrine. (H) *SS-*Formoterol. (I) *SR-*Droxidopa. The ligand is depicted in green, while the interacting residues of *Lam*ARG are represented in purple. Hydrogen bonds are illustrated with green dotted lines, π-π stacking interactions with purple dotted lines, cation-π interactions with orange dotted lines, and ion-dipole interactions with gray dotted lines. Residues interacting through hydrophobic interactions with the inhibitors are also displayed in the figure.

**
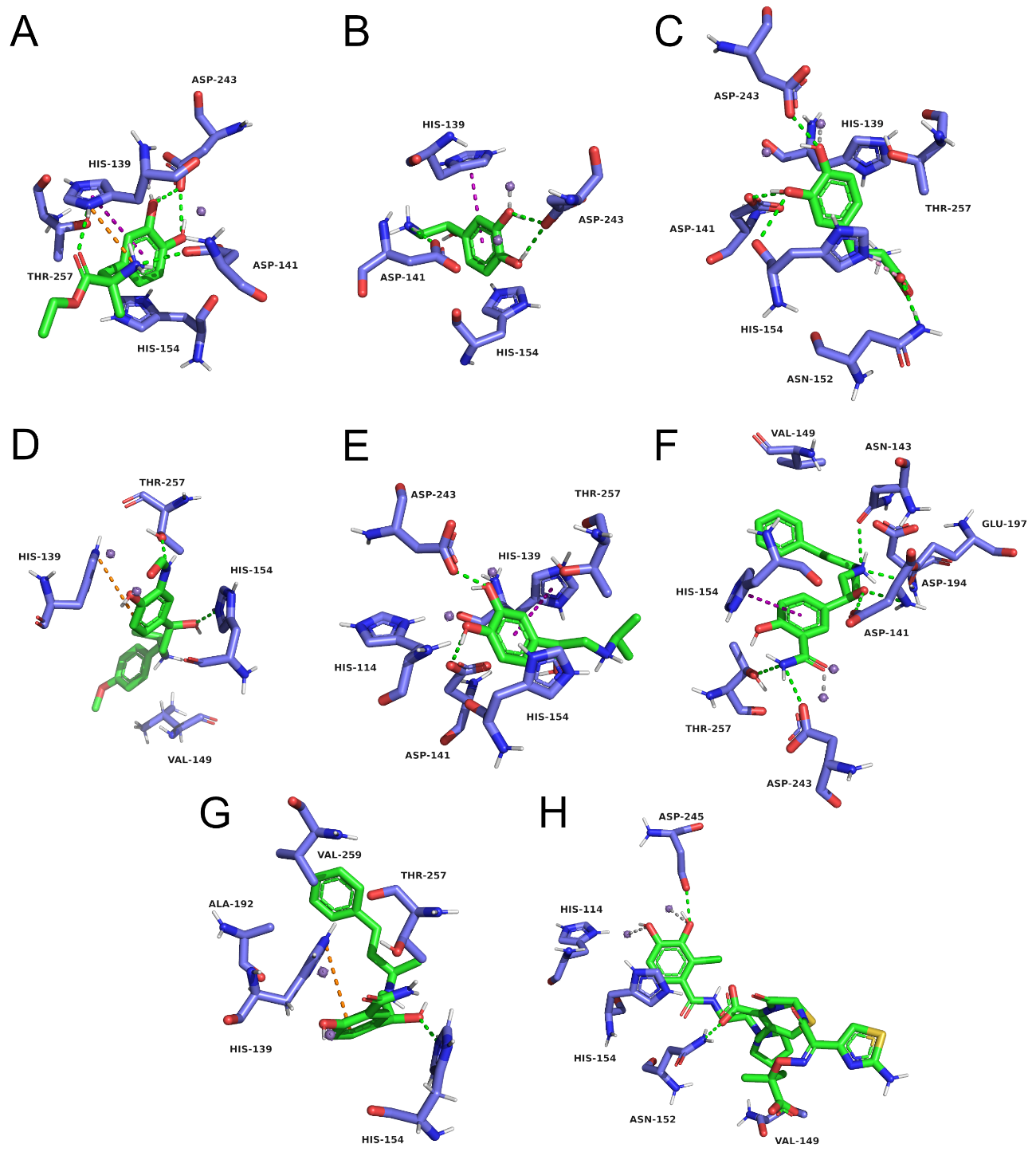
**

**Figure S5** **Predicted binding modes for potential *Lam*ARG inhibitors (Part 2).** (A) Methyldopate. (B) Norepinephrine. (C) Levonordefrin. (D) *RR*-Arformoterol. (E) *S*-Isoproterenol. (F) *SS*-Labetalol. (G) *SR-*Labetalol. (H) Cefiderocol. The ligand is depicted in green, while the interacting residues of *Lam*ARG are represented in purple. Hydrogen bonds are illustrated with green dotted lines, π-π stacking interactions with purple dotted lines, cation-π interactions with orange dotted lines, and ion-dipole interactions with gray dotted lines. Residues interacting through hydrophobic interactions with the inhibitors are also displayed in the figure.

**
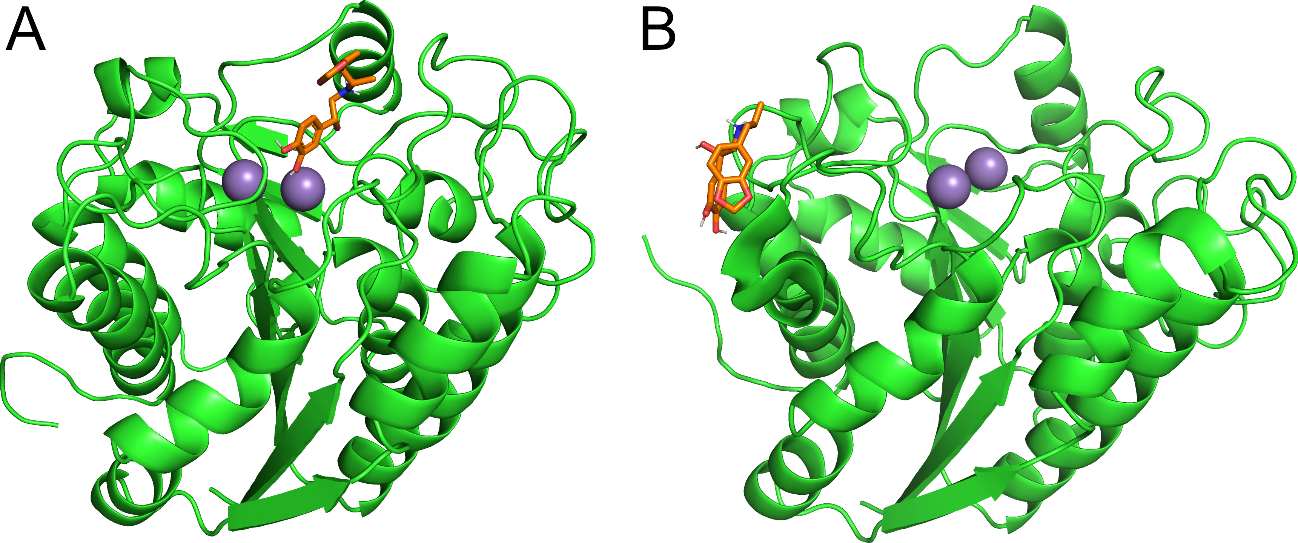
**

**Figure S6 Three-dimensional structure of the *Lam*ARG-Protokylol complex before and after molecular dynamics (MD) simulations.** (A) Structure of the complex at the beginning of the simulation, corresponding to the configuration predicted by molecular docking. (B) Structure at the end of the 300 ns simulation.
